# Clec7a Worsens Long‐Term Outcomes after Ischemic Stroke by Aggravating Microglia‐Mediated Synapse Elimination

**DOI:** 10.1002/advs.202403064

**Published:** 2024-08-01

**Authors:** Hanxi Wan, Mengfan He, Chun Cheng, Kexin Yang, Huanghui Wu, Peilin Cong, Xinwei Huang, Qian Zhang, Yufei Shi, Ji Hu, Li Tian, Lize Xiong

**Affiliations:** ^1^ Shanghai Key Laboratory of Anesthesiology and Brain Functional Modulation Translational Research Institute of Brain and Brain‐Like Intelligence Clinical Research Center for Anesthesiology and Perioperative Medicine Department of Anesthesiology and Perioperative Medicine Shanghai Fourth People's Hospital School of Medicine Tongji University Shanghai 200434 China; ^2^ School of Life Science and Technology ShanghaiTech University Shanghai 201210 China

**Keywords:** Clec7a, ischemic stroke, microglia, phagocytosis, synapses

## Abstract

Ischemic stroke (IS) is a leading cause of morbidity and mortality globally and triggers a series of reactions leading to primary and secondary brain injuries and permanent neurological deficits. Microglia in the central nervous system play dual roles in neuroprotection and responding to ischemic brain damage. Here, an IS model is employed to determine the involvement of microglia in phagocytosis at excitatory synapses. Additionally, the effects of pharmacological depletion of microglia are investigated on improving neurobehavioral outcomes and mitigating brain injury. RNA sequencing of microglia reveals an increase in phagocytosis‐associated pathway activity and gene expression, and C‐type lectin domain family 7 member A (Clec7a) is identified as a key regulator of this process. Manipulating microglial Clec7a expression can potentially regulate microglial phagocytosis of synapses, thereby preventing synaptic loss and improving neurobehavioral outcomes after IS. It is further demonstrat that microglial Clec7a interacts with neuronal myeloid differentiation protein 2 (MD2), a key molecule mediating poststroke neurological injury, and propose the novel hypothesis that MD2 is a ligand for microglial Clec7a. These findings suggest that microglial Clec7a plays a critical role in mediating synaptic phagocytosis in a mouse model of IS, suggesting that Clec7a may be a therapeutic target for IS.

## Graphical abstract

In this study, Clec7a, a pattern‐recognition receptor, is identified as a key trigger of ischemic stroke. Clec7a is upregulated in microglia after ischemic stroke. In mice, knockout of microglial Clec7a decreases the engulfment of synapses, increases the number of synapses, and thereby significantly mitigates stroke‐induced neurological deficits. Therefore, Clec7a may serve as a potential therapeutic target for ischemic stroke.

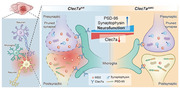

## 1 Introduction

Approximately 12.2 million cases of ischemic stroke (IS) occur worldwide each year. Globally, stroke is the second leading cause of death (6–11% of all deaths) and the third leading cause of disability.^[^ ^1^ ^]^ Despite substantial progress in the treatment of stroke, severe long‐term neurological impairments, including sensory impairment, decreased motor ability, and diminished cognitive flexibility, continue to occur in stroke patients. Microglia, the primary immune cells in the central nervous system (CNS), play essential roles in maintaining neuronal stability, regulating neurotransmitter release, and participating in neuronal repair and regeneration. Recently, increasing evidence has shown that microglia play a key role in the onset and progression of IS. Research using CX3CR1^CreER^:R26^iDTR^ mice to deplete microglia with tamoxifen and diphtheria toxin revealed a significant decrease in ischemic infarct volume and improved motor function poststroke.^[^ ^2^ ^]^ However, some studies have indicated that microglia depletion worsens ischemic injuries, while repopulation offers neuroprotection.^[^ ^3^ ^]^

In contrast, microglia function as professional phagocytes in the CNS, clearing necrotic brain tissue and toxic cellular remnants and decreasing proinflammatory factor levels through phagocytosis, which is generally considered beneficial.^[^ ^4^, ^5^, ^6^, ^7^, ^8^ ^]^ However, recent research has indicated that excessive microglial phagocytosis, particularly when viable brain cells, primarily neurons, are mistakenly engulfed, can exacerbate neuronal loss and contribute to delayed brain atrophy and neurodegeneration.^[^ ^9^, ^10^, ^11^ ^]^ Synapses, crucial structures of neurons, play a pivotal role in neuronal function. Synaptic formation and remodeling occur continuously throughout the brain and are essential for the function of neural circuits and neurotransmitter dynamics.^[^ ^12^, ^13^ ^]^ During brain development, microglia clear excess or functionally weak synapses through phagocytosis, ensuring the formation of correct neural circuits.^[^ ^14^ ^]^ However, abnormal phagocytosis during this period may lead to behavioral disorders in adulthood, such as Alzheimer's disease (AD), autism, schizophrenia, and attention‐deficit hyperactivity disorder.^[^ ^15^, ^16^, ^17^, ^18^ ^]^ Microglia‐mediated synapse loss is an early and key feature underlying circuit dysfunction in many neurodegenerative diseases, including AD and related neurodegenerative diseases.^[^ ^9^, ^19^, ^20^ ^]^ Research has demonstrated that after IS, reactive microglia and astrocytes can phagocytose synapses via MEGF10 and MERTK‐related signaling pathways.^[^ ^20^ ^]^ Thus, increased microglial phagocytosis of synapses after IS may be an important factor in triggering poststroke neurological deficits.

In the CNS, microglia rely on immune molecules to recognize inappropriate or unnecessary synapses.^[^ ^21^, ^22^ ^]^ The innate immune molecules involved in this process include components of the complement cascade, C1q and C3, which are often referred to as “eat‐me” signals because they promote phagocytosis by binding to unnecessary or harmful substances.^[^ ^10^, ^23^, ^24^ ^]^ In the brain, C1q and C3 are found in developing synapses and facilitate microglial engulfment of synapses, a process dependent on the microglial C3 receptor (CR3).^[^ ^15^, ^24^ ^]^ Mice deficient in these molecules exhibit impaired synaptic refinement, while C1q and CR3 knockout mice have excess synapses in adulthood. We identified a member of the C‐type lectin receptor family, Clec7a, as a receptor involved in microglial phagocytosis. We observed that Clec7a is required for synaptic engulfment by microglia after IS. Clec7a has been shown to regulate phagocytosis by macrophages in multiple peripheral tissues. Moreover, Clec7a is upregulated in microglia during neurodegeneration and is an important receptor for microglial activation in response to AD pathology. Furthermore, Clec7a signals through spleen tyrosine kinase (SYK) to enhance the phagocytosis of Aβ.^[^ ^25^ ^]^

Thus, we aimed to demonstrate that microglia mediate synapse loss and neurological function impairment after IS. Our research revealed the critical role of microglial Clec7a in long‐term neurological deficits following IS. Regulating the expression of microglial Clec7a may be an effective strategy for promoting long‐term neurological function recovery after IS.

## 2 Results

### 2.1 Phagocytosis of Synapses by Microglia after IS

To determine how microglia respond to IS, we assessed microglial activation using immunofluorescence (IF) staining for the microglial marker Iba‐1 in the cortex (Figure S1A, Supporting Information) and hippocampus (Figure S1F, Supporting Information) at 1, 3, 7, 14, and 28 days after transient middle cerebral artery occlusion (tMCAO). Microglia in the poststroke cortex exhibited an activated phenotype characterized by an increased cell number, enlarged cell bodies, and fewer complex branches (Figure S1B–E, Supporting Information). Notably, microglial activation was also found in the hippocampus (Figure S1G–J, Supporting Information), which is another susceptible region known to be affected by IS.^[^ ^26^, ^27^ ^]^ We also detected neurological deficits after IS (Figure S2A,B, Supporting Information), as assessed by the modified neurological severity score (mNSS) (Figure S2C, Supporting Information), rotarod test (Figure S2D, Supporting Information), adhesive removal test (Figure S2E,F, Supporting Information), and water maze test (Figure S2G–L, Supporting Information). Overall, these findings showed that tMCAO induced microglia activation and neurological deficits in mice. Notably, the activation of microglia in both the cortex and hippocampus was greatest at 7 days after IS. Therefore, our research mainly focused on investigating microglia 7 days after IS.

**Figure 1:**
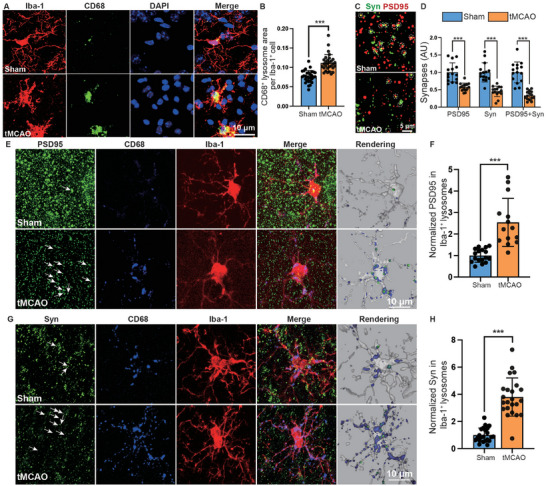
Synaptic phagocytosis by microglia after ischemic stroke. A) CD68^+^ lysosome content (green) in Iba‐1^+^ microglia (red) in the ischemic penumbra of sham and tMCAO mice. Scale bars, 10 µm. B) Quantification of the lysosomal area relative to the microglial area. C) Representative single‐plane images of PSD95 and synaptophysin (Syn) (excitatory synapses) in the ischemic penumbra of sham and tMCAO mice. Dotted circles highlight colocalized synapses. Scale bars, 5 µm. D) Quantification of excitatory synapses. E‐H. Confocal images and quantification of synaptic puncta engulfed by microglia showing the colocalization of PSD95 or Syn with CD68 within microglia in the ischemic penumbra of sham and tMCAO mice. The white arrows indicate PSD95^+^CD68^+^ E) or Syn^+^CD68^+^ G) puncta within microglia. Mice aged 8–10 weeks were used for the experiments shown in this figure. Scale bars, 10 µm. Statistics were derived from 18 slices, *n* = 6 mice for each group. In B, D, F, and H, significance was calculated using two‐tailed unpaired Student's *t*‐test. Data are presented as mean ± SD. ***p < 0.001.

To determine whether activated microglia develop phagocytic capabilities after IS, we examined the expression of the phagocytosis markers CD68 and Iba‐1 (**Figure** **1**A; Figure S3A, Supporting Information). The results revealed a significant increase in the number of CD68^+^ lysosomes within microglia following IS, and the phagocytic ability of microglia is strongest at 7 days post‐IS and persisted for 28 days (Figure 1B; Figure S3B, Supporting Information). Furthermore, confocal images of Synaptophysin^+^ (Syn) presynapses and PSD95^+^ postsynapses were examined to determine the quantity of synapses (Figure 1C). The analysis demonstrated a reduction in synaptic density after IS (Figure 1D). Next, we quantified the extent to which PSD95 and Syn‐immunoreactive synaptic puncta were internalized within CD68^+^ Iba‐1^+^ microglial lysosomes (Figure 1E,G), and the results showed an increase in the engulfment of synapses by microglia (Figure 1F,H).

**Figure 2:**
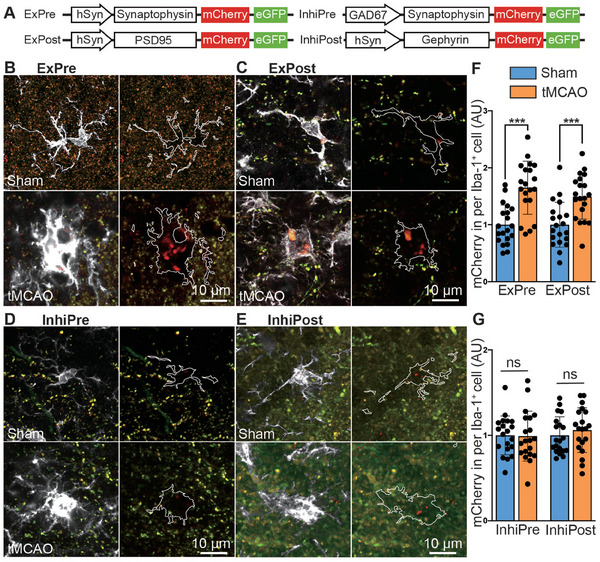
Microglia eliminate excitatory synapses after ischemic stroke. A) Schematic illustration of reporters for synapse elimination. B–E) Representative images of sham and tMCAO microglia (Iba‐1^+^, white) with only mCherry puncta (red) derived from ExPre (B), ExPost (C), InhiPre (D) or InhiPost (E) reporters in the ischemic penumbra. Microglia are highlighted with white outlines. F,G) Quantification of the volume of synapses containing mCherry alone within microglia. Scale bars, 10 µm. ExPre, excitatory presynapse; ExPost, excitatory postsynapse; InhiPre, inhibitory presynapse; InhiPost, inhibitory postsynapse. Mice aged 8–10 weeks were used for the experiments shown in this figure. The statistical data were derived from 20 slices, *n* = 4 mice per group. AUs, arbitrary units. In F and G significance was calculated using two‐tailed unpaired Student's *t*‐test. Data are presented as mean ± SD. ***p < 0.001; ns indicates no significant difference.

To delineate the synaptic subtypes engulfed within the ischemic brain, we administered four novel fluorescent phagocytosis reporters to the striatal region of different mice.^[^ ^28^ ^]^ ExPre encodes Synaptophysin‐mCherry‐eGFP, which is driven by the hSyn promoter and targets presynaptic structures of excitatory synapses. The hSyn promoter also drives the expression of PSD95‐mCherry‐eGFP in excitatory synapses (ExPost), and Gephyrin‐mCherry‐eGFP in inhibitory synapses (InhiPost). InhiPre encodes Synaptophysin‐mCherry‐eGFP, which is driven by the Gad67 promoter to localize to inhibitory synapses (**Figure** **2**A). eGFP and mCherry exhibited different sensitivities to the lysosomal environment. eGFP fluorescence was attenuated in lysosomes due to degradation by lysosomal hydrolases, whereas mCherry fluorescence remained stable. Activated microglia from mice subjected to tMCAO showed increased engulfment of excitatory pre‐ and postsynaptic elements (Figure 2B,C,F; Figure S4, Supporting Information) without a clear change in the engulfment of inhibitory pre‐ and postsynaptic elements (Figure 2D,E,G; Figure S4, Supporting Information).

Collectively, these findings indicate the natural turnover of synapses after IS and show that microglia play a significant role in this process by consistently engulfing excitatory synapses.

### 2.2 Pharmacological Ablation of Microglia Alleviates Long‐Term Impairment of Neurological Function

**Figure 3:**
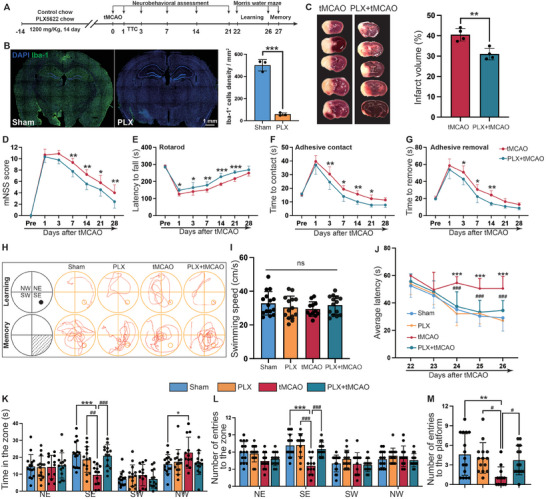
Microglia are essential for long‐term neurological dysfunction and cognitive impairment after ischemic stroke. A) Overview of the experimental timeline. PLX5622 treatment was initiated 2 weeks before surgery (after surgery, all mice returned to a normal diet), neurobehavioral assessments were conducted 1, 3, 7, 14, 21, and 28 days after surgery, and Morris water maze experiments were performed 22–27 days after surgery. B) Representative confocal images and quantification of microglial density in the whole brain confirming the loss of Iba‐1^+^ microglia (green) in PLX5622‐treated mice after 14 days. Scale bars, 1 mm. *n* = 3 mice per group. C) Representative images of TTC‐stained coronal brain sections (left) from mice after tMCAO. The right panel shows the quantification of the relative infarct volume. *n* = 4 mice per group. D–G) Long‐term neurological dysfunction after treatment with PLX5622 was assessed with the mNSS (D), rotarod test (E), adhesive contact test (F), and adhesive removal test (G). *n* = 9 mice per group. H–M) The results of the Morris water maze test: schematic diagram and representative navigation trajectories for learning and memory (H); swimming speed (I); summary data for escape latency (J); time spent in the four zones (K); number of entries into the four zones (L); and number of entries onto the platform (M). *n* = 12–14 mice per group. Mice aged 8–10 weeks were used for the experiments shown in this figure. Significance was calculated using either two‐tailed unpaired Student's *t*‐test (B‐C), one‐way ANOVA (I, M), or two‐way ANOVA (D‐G, J‐L), followed by Tukey's multiple comparisons test. Data are presented as mean ± SD. *p < 0.05, **p < 0.01, and ***p < 0.001 versus Sham; ^#^ *p *< 0.05, ^##^ *p *< 0.01, and ^###^ *p *< 0.001 versus tMCAO, ns indicates no significant difference.

Further investigation was needed to determine the role of microglia in long‐term neurological impairment after stroke; therefore, the colony‐stimulating factor 1 receptor (CSF1R) inhibitor PLX5622 was used to deplete microglia in mice (**Figure** **3**A). A notable decrease in the quantity of microglia was detected in mice fed food containing PLX5622 compared with sham mice (Figure 3B). TTC staining revealed a markedly reduced infarct volume in PLX5622‐treated mice compared with sham mice after IS (Figure 3C). As important indicators of neurological recovery after IS, sensorimotor and motor functions were measured with the mNSS (Figure 3D), rotarod test (Figure 3E), and adhesive removal test (Figure 3F,G). The results demonstrated that, compared with sham mice, PLX5622 treated mice exhibited improved sensorimotor and motor function, with significant improvements observed ≈3 days poststroke. PLX5622 treatment also ameliorated poststroke deficits in spatial cognitive function after IS (Figure 3H), as evidenced by a reduced latency to find the hidden platform (improved spatial learning; Figure 3J), increased time spent in the target zone (improved memory retention; Figure 3K), increased number of entries into the target zone (improved memory retention; Figure 3L), and increased number of entries into the platform region (improved memory retention; Figure 3M) after the platform was removed in the Morris water maze. There was no difference in swimming speed among the various treatment groups, as shown in Figure 3I, suggesting similar swimming abilities across the groups. Taken together, these data revealed that depleting microglia via PLX5622 rescues long‐term neurological function after IS.

### 2.3 RNA‐seq Analysis of Microglia in IS

**Figure 4:**
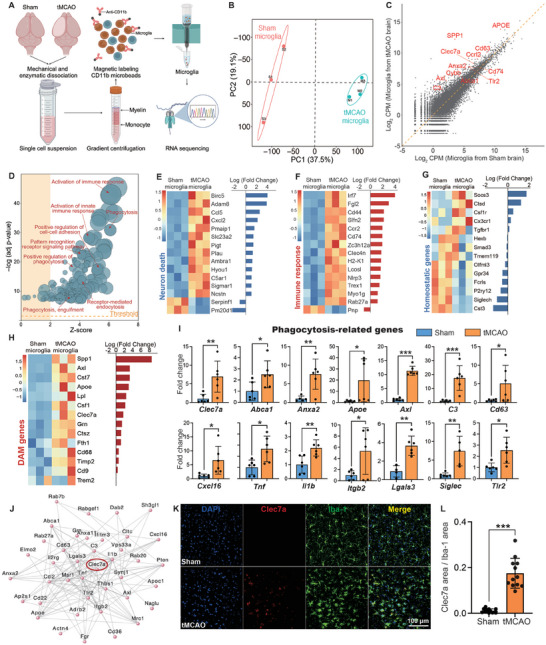
Transcriptome sequencing reveals microglial responses to ischemic stroke. A) Schematic of RNA‐seq analysis of microglia isolated from the ischemic brain 7 days after tMCAO and from sham mice, *n* = 3 per group, each n consists of 30 mice. B) Principal component analysis (PCA) plot. The two groups of samples were separated when plotting the first (PC1) versus the second component (PC2). C) Scatterplot showing the comparison of log_2_CPM (counts per million) in sham microglia versus tMCAO microglia; red dots represent DAM genes. The orange dashed line indicates a fold change of 1. D) GO analysis of DEGs identified key biological processes in tMCAO microglia compared with sham microglia, which are graphically displayed in bubble plots according to Z scores and significance (log10[adjusted *p* value]). E–H) Heatmap showing genes related to neuronal death (E), immune response (F), homeostatic state (G) and DAM state (H) in tMCAO microglia versus sham microglia. I) qRT‒PCR analysis showing the expression of phagocytosis‐related genes in isolated microglia from the ischemic penumbra of tMCAO and sham mice, *n* = 6 per group, each n consists of 3 mice. J) Protein‒protein interaction (PPI) network for microglial phagocytosis‐related genes. K,L). Representative single‐plane images (K) and quantitative analyses (L) of Clec7a (red) in Iba‐1^+^ microglia (green) in the ischemic penumbra from sham and tMCAO mice. Scale bars, 100 µm. Mice aged 8–10 weeks were used for the experiments shown in this figure. Statistics were derived from 12 slices, *n* = 4 mice per group. In I and L significance was calculated using two‐tailed unpaired Student's *t*‐test. Data are presented as mean ± SD. *p < 0.05, **p < 0.01, and ***p < 0.001.

To further investigate the role of microglia in IS, microglia were isolated for RNA‐seq. Briefly, microglia were isolated from 8‐week‐old sham or tMCAO mice via magnetic separation (**Figure** **4A**). Principal component analysis (PCA) revealed that the overall transcriptomic profiles were markedly changed in microglia after IS compared to those in microglia from sham mice (Figure 4B). Scatterplots of the normalized average gene expression in sham microglia compared with tMCAO microglia (Figure 4C,H) revealed that several damage‐associated microglia (DAM) genes (*Clec7a, Spp1, Axl, APOE*, etc.) were upregulated in microglia from mice subjected to tMCAO. The specific gene expression patterns of microglia from mice subjected to tMCAO demonstrated enrichment of genes involved in immunoregulation, particularly those involved in activating phagocytosis (Figure 4D).

Microglia from the tMCAO group displayed significant upregulation of neuronal death‐related genes, such as *Ccl5, Cxcl2, Slc23a2*, and *Ncstn* (Figure 4E). Genes encoding immune response proteins, such as *Ccr2, Cd44, Irf7, H2‐K1*, and *Trex1*, were also upregulated (Figure 4F). No significant differences in the expression of homeostatic microglial genes, such as *Tmem119*, *Cx3cr1*, and *P2ry12*, were observed between the two groups (Figure 4G). On the contrary, DAM genes, such as *Spp1*, *Axl*, *Cst7*, *Apoe*, *Lpl*, *Csf1*, and *Clec7a*, were upregulated (Figure 4H). Notably, the expression of genes related to phagocytosis, including *Clec7a, Abca1, Anxa2, Apoe*, and *Axl*, were upregulated in microglia from mice subjected to tMCAO (Figure 4I). Clec7a, a key member of the C‐type lectin receptor family,^[^ ^25^ ^]^ was significantly upregulated, suggesting its crucial role in the microglial response to IS and making it a critical target for further investigation. The critical involvement of Clec7a was identified via protein‐protein interaction analysis of these phagocytosis‐related genes (Figure 4J). Further study revealed a notable increase in the expression of Clec7a in microglia after IS (Figure 4K,L; Figure S5, Supporting Information).

### 2.4 Clec7a‐Mediated Microglial Engulfment of Synapses after IS

We investigated whether microglial phagocytosis of synapses is mediated by microglial Clec7a. Initially, we altered Clec7a expression in BV2 cells and subsequently adapted an established synaptosome engulfment assay to quantify the engulfment of pHrodo‐conjugated synaptosomes. When synaptosomes were administered, Clec7a‐knockdown BV2 cells engulfed fewer synapses than control BV2 cells (Figures S6A and S7A,C, Supporting Information). Conversely, compared with control BV2 cells, Clec7a‐overexpressing BV2 cells exhibited an increased level of synapse engulfment (Figures S6B and S7B,D, Supporting Information). Taken together, these results suggested that microglial Clec7a expression was associated with synaptic phagocytosis.

**Figure 5:**
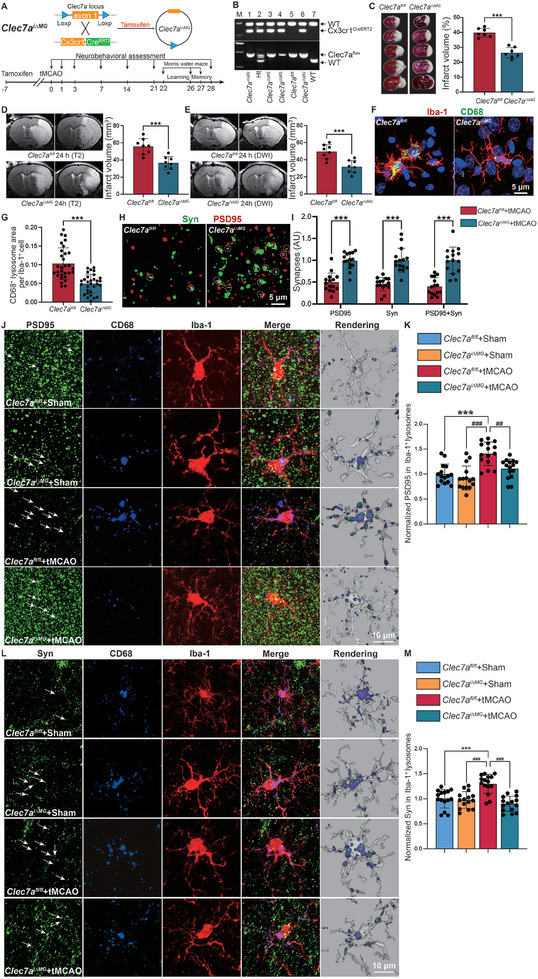
Clec7a promotes microglia‐mediated synaptic phagocytosis after ischemic stroke. A) Schematic of the experimental design. B) PCR genotyping showed that *Clec7a^i∆MG^* (lanes 1, 3, 4, and 6) and WT littermate control (lane 7) mice were successfully generated by crossing *Cx3cr1^CreERT2^* transgenic mice with *Clec7a^fl/fl^* mice. Heterozygotes (Ht) that contained both Clec7a WT and *Clec7a^flox^* bands are shown. C) Representative photographs of TTC staining (left) after tMCAO and quantification of the relative infarct volume (right). *n* = 7 mice per group. D) T2 images at 24 h after the onset of ischemia in both groups. *n* = 8 mice per group. E) DW images of both groups at 24 h after ischemic stroke. *n* = 8 mice per group. F) CD68^+^ lysosome content (green) in Iba‐1^+^ microglia (red) in the ischemic penumbra from *Clec7a^i∆MG^* and *Clec7a^fl/fl^* mice subjected to tMCAO. Scale bars, 5 µm. G) Quantification of the lysosomal area relative to the microglial area. H) Representative single‐plane images of Syn and PSD95 staining in the ischemic penumbra. Dotted circles highlight colocalized synapses. Scale bars, 5 µm. I) Quantification of excitatory synapses. J–M) Confocal images and quantification of synaptic puncta engulfed by microglia showing the colocalization of PSD95 (J, K) or Syn (L, M) with CD68 within microglia in the ischemic penumbra from sham and tMCAO mice. The white arrows indicate PSD95^+^CD68^+^ (J) or Syn^+^CD68^+^ (L) puncta within microglia. Scale bars, 10 µm. Statistics were derived from 18 slices, *n* = 6 mice per group. Mice aged 8–10 weeks were used for the experiments shown in this figure. Significance was calculated using either two‐tailed unpaired Student's *t*‐test (C–E, G, and I) or one‐way ANOVA, Tukey's multiple comparisons test (K and M). Data are presented as mean ± SD. ***p < 0.001 versus Sham; ^##^ *p *< 0.01 and ^###^ *p *< 0.001 versus tMCAO.

Based on the aforementioned in vitro findings regarding the regulation of phagocytic synapses in microglia by Clec7a, we investigated whether Clec7a also plays a role in the regulation of microglial synaptic phagocytosis in vivo. We crossed *Clec7a^fl/fl^* mice with *Cx3cr1^CreERT2^* mice, enabling the inducible deletion of *Clec7a* in *Cx3cr1*‐positive cells via tamoxifen administration. We referred to these mice as *Clec7a^i∆MG^* mice, with *Clec7a^fl/fl^* mice serving as controls (**Figure** **5**A,B; Figure S8, Supporting Information). *Clec7a^i∆MG^* mice exhibited a reduced infarct size compared with *Clec7a^fl/fl^* mice according to TTC staining, diffusion‐weighted imaging (DWI), and T2‐weighted spin‒echo imaging (T2) at 1 day after tMCAO (Figure 5C–E). There was a decrease in the phagocytosis of lysosomes (Figure 5F,G) by microglia, an increase in the number of synapses (Figure 5H,I) in the ischemic brain in *Clec7a^i∆MG^* mice, and a decrease in the number of synapses phagocytosed by microglia after IS (Figure 5J–M).

### 2.5 Clec7a has a Significant Impact on Neurological Damage after IS

**Figure 6:**
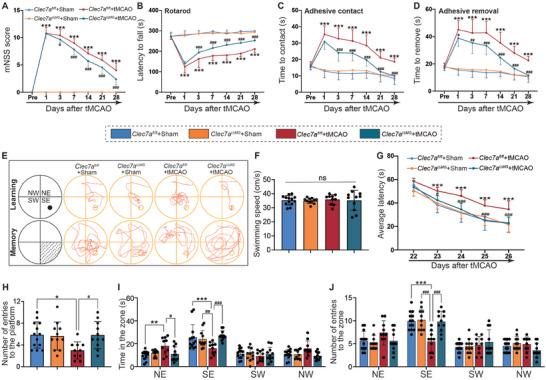
Inducible knockdown of microglial Clec7a rescues impaired neurological function after ischemic stroke. A–D) Long‐term neurological dysfunction after tMCAO was assessed with the mNSS (A), rotarod test (B), adhesive contact test (C), and adhesive removal test (D). E) Schematic diagram and representative navigation trajectories for learning and memory. F) The swimming speed is presented. G) The average escape latencies. H) The number of entries onto the platform. I) The duration in each zone. J) The number of entries into each zone. *n* = 10‐13 mice per group. Mice aged 8–10 weeks were used for the experiments shown in this figure. Significance was calculated using either one‐way ANOVA (F and H) or two‐way ANOVA (A‐D, G, and I,J), followed by Tukey's multiple comparisons test. Data are presented as mean ± SD. *p < 0.05, **p < 0.01, and ***p < 0.001 versus Sham; ^#^ *p *< 0.05, ^##^ *p *< 0.01, and ^###^ *p *< 0.001 versus tMCAO; ns indicates no significant difference.

To observe whether Clec7a knockout could improve neurological function after IS, we measured sensorimotor and motor function by the mNSS (**Figure** **6**A), rotarod test (Figure 6B), and adhesive removal test (Figure 6C,D). Compared with *Clec7a^fl/fl^* mice, *Clec7a^i∆MG^* mice exhibited improved sensorimotor and motor functions after IS. In the Morris water maze (Figure 6E), the mice exhibited similar swimming velocities (Figure 6F). During the training phase, the latency to find the target platform was significantly shorter in *Clec7a^i∆MG^* mice than in *Clec7a^fl/fl^* mice after IS (Figure 6G). The results of the memory test indicated that the duration of time spent in the target zone following the removal of the platform (Figure 6I), as well as the the number of entries into both the target zone (Figure 6J) and the platform (Figure 6H), increased in *Clec7a^i∆MG^* mice after IS. Therefore, these findings demonstrated that the suppression of microglial Clec7a improved long‐term neurological function following IS by inhibiting microglial phagocytosis of synapses.

### 2.6 Clec7a Affects the Activity of Ischemic Penumbra Neurons after IS

**Figure 7:**
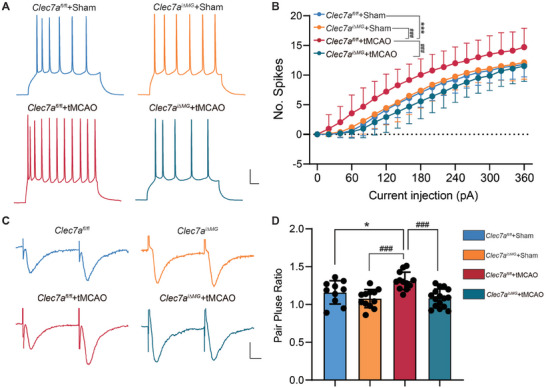
Microglial Clec7a knockout restores neuronal activity and synaptic transmission after ischemic stroke. A) Representative traces of the action potentials recorded from ischemic penumbra neurons in +180 pA current injection of *Clec7a^fl/fl^* and *Clec7a^i∆MG^* mice subjected to tMCAO and sham operation. Scale bar, 20 mV, 50 ms. B) Statistics showed that as the injection current increased (from 0 to +360 pA), the number of spikes was significantly increased in tMCAO *Clec7a^fl/fl^* mice compared to tMCAO *Clec7a^i∆MG^* and sham mice (*Clec7a^fl/fl^* group, *n* = 16 neurons in 3 mice, *Clec7a^i∆MG^* group, *n* = 16 neurons in 3 mice; *Clec7a^fl/fl^* + tMCAO group, *n* = 22 neurons in 5 mice, *Clec7a^i∆MG^* + tMCAO group, *n* = 24 neurons in 6 mice). C) Representative PPR traces of *Clec7a^fl/fl^* and *Clec7a^i∆MG^* mice subjected to tMCAO and sham operation after two consecutive stimuli. Scale bar, 100pA, 10 ms. D) Statistical analysis showed that the ratios of tMCAO *Clec7a^fl/fl^* mice was increased compared to tMCAO *Clec7a^i∆MG^* and sham mice (*Clec7a^fl/fl^* group, *n* = 10 neurons in 3 mice, *Clec7a^i∆MG^* group, *n* = 12 neurons in 3 mice; *Clec7a^fl/fl^* + tMCAO group, *n* = 12 neurons in 5 mice, *Clec7a^i∆MG^* + tMCAO group, *n* = 16 neurons in 6 mice). Statistical significance was determined by two‐way ANOVA (B) and one‐way ANOVA (D) followed by Tukey's multiple comparisons test. Data are presented as mean ±  SD. *p < 0.05 and ***p < 0.001 versus Sham; ^###^ *p *< 0.001 versus tMCAO.

To test whether Clec7a regulates the excitability of neurons in tMCAO and sham mice, we tested neuronal firing in the ischemic penumbra of brain slices. We found that Clec7a knockout had no effect on the neuronal excitability in sham‐operated *Clec7a^fl/fl^* and *Clec7a^i∆MG^* mice. However, we detected increased action potentials evoked by current injection in *Clec7a^fl/fl^* mice after IS, and the hyperexcitability of neurons was restored in *Clec7a^i∆MG^* mice (**Figure** **7**A,B). Our previous observations showed that microglial Clec7a mediates microglial phagocytosis of synapses. We then investigated changes in synaptic transmission induced by lack of Clec7a in microglia after IS. Our recordings showed that the pair pulse ratio (PPR) in *Clec7a^fl/fl^* slices was significantly increased compared with sham‐operated mice and that Clec7a knockout restored normal synaptic connectivity (Figure 7C,D). In contrast, IS induced a large amount of neurodegeneration as revealed by FJC staining. There was no significant difference between sham‐operated *Clec7a^fl/fl^* and *Clec7a^i∆MG^* mice. The number of FJC^+^ cells increased in *Clec7a^fl/fl^* mice after IS, and reduced in *Clec7a^i∆MG^* IS mice (Figure S9, Supporting Information).

These data indicated that knocking out of microglial Clec7a reduced neuronal excitability, neurodegeneration, and rescued synaptic transmission after IS.

### 2.7 Clec7a Interacts with MD2 after Ischemic Stroke

**Figure 8:**
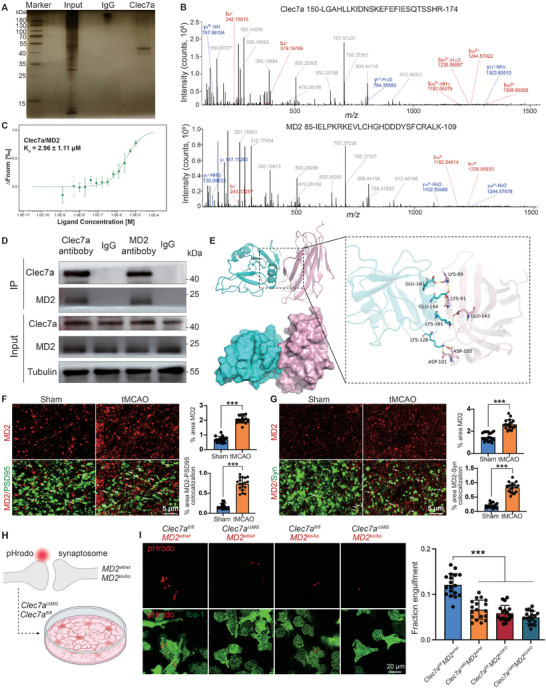
Clec7a interacts with MD2 in the ischemic brain. A) Immunoprecipitates were subjected to SDS‐PAGE and silver staining. B) Annotated LC–MS/MS spectra of 150‐LGAHLLKIDNSKEFEFIESQTSSHR‐174 from Clec7a and 85‐IELPKRKEVLCHGHDDDYSFCRALK‐109 from MD2 in anti‐Clec7a immunoprecipitates from tMCAO mice. C) Normalized binding curve of Clec7a and MD2. The binding curve yielded a Kd of 2.96 ± 1.11 µm. The concentration of Clec7a was kept constant at 50 nm while the MD2 concentration was varied from 10 µM to 1.2 nM. *n *= 3 per group. D) Endogenous Clec7a was coimmunoprecipitated with MD2 after ischemic stroke. The experiments were repeated independently three times. E) The interaction between Clec7a (cyan) and MD2 (pink). The yellow dashed line indicates hydrogen bonding. F) Representative images and analysis of the ischemic penumbra showing the colocalization of MD2 with PSD95, as indicated by the white arrowheads; scale bars, 5 µm. G) Representative images and analysis of the ischemic penumbra showing the colocalization of MD2 with Syn as indicated by the white arrowheads; scale bars, 5 µm. H) Schematic illustrating the in vitro phagocytosis assay in which primary microglia isolated from *Clec7a^i∆MG^* or *Clec7a^fl/fl^* mice phagocytosed *MD2^ko/ko^* or *MD2^wt/wt^* synaptosomes tagged with pHrodo. I) Representative images of cultured *Clec7a^i∆MG^* or *Clec7a^fl/fl^* microglia engulfing pHrodo‐conjugated synaptosomes (top: engulfed pHrodo; bottom: merged image of Iba‐1 and pHrodo). Scale bars, 20 µm. Statistics were derived from 18 slices, *n* = 6 per group. Mice aged 8–10 weeks were used for the experiments shown in this figure. Significance was calculated using either two‐tailed unpaired Student's *t*‐test (F and G) or one‐way ANOVA, Tukey's multiple comparisons test (I). Data are presented as mean ± SD. ***p < 0.001.

To further investigate the specific mechanisms of microglial Clec7a phagocytosis, we performed liquid chromatography with tandem mass spectrometry (LC–MS/MS) on proteins from the penumbra region to explore which proteins interact with Clec7a. We found that the extracellular peptide of Clec7a (150‐LGAHLLKIDNSKEFEFIESQTSSHR‐174) interacts with myeloid differentiation protein 2 (MD2, 85‐IELPKRKEVLCHGHDDDYSFCRALK‐109) (**Figure** **8**A,B). Our previous study demonstrated that neuronal MD2, a critical molecule associated with neural injury, is upregulated following IS.^[^ ^29^ ^]^ Next, we examined the interaction between Clec7a and MD2 using microscale thermophoresis (MST), and the results confirmed the high binding affinity between the two proteins (Kd = 2.96 µm) (Figure 8C). On the other hand, immunoprecipitation (CoIP) assays demonstrated the coimmunoprecipitation of endogenous Clec7a with MD2 (Figure 8D).

To examine the interaction between Clec7a and MD2 at the molecular level, we performed molecular dynamics (MD) simulations using the force field ff14SB and the crystal structures of Clec7a (PDB code: Q6QLQ4) and MD2 (PDB code: Q9JHF9), as shown in the cartoon diagram. In the Clec7a/MD2 complex, Glu‐167, Glu‐164, Lys‐161, and Lys‐128 on Clec7a form hydrogen bonds with Lys‐89, Lys‐91, Glu‐143, Asp‐100, and Asp‐101 on MD2, respectively (Figure 8E). The root‐mean‐square deviation (RMSD) values of the MD simulations indicated that the binding of Clec7a to MD2 was stable (Figure S10A, Supporting Information). The RMSF values for most protein regions fell within the range of 2 Å, suggesting that the protein's primary structure exhibited a high degree of rigidity. Consequently, it can be inferred that the protein displays limited flexibility within the complex system, thereby forming the foundation for protein–protein interactions (Figure S10B, Supporting Information). We also monitored the change in the number of hydrogen bonds formed within the protein–protein complex over time via MD simulations. The number of hydrogen bondsestablished between small molecules and proteins throughout the simulation varied between 0 and 11, demonstrating the significance of hydrogen bonding in the interaction between Clec7a and MD2 (Figure S10C, Supporting Information). Then MM‐GBSA energy decomposition was used to identify the top 20 amino acids in Clec7a contributing to the Clec7a/MD2 binding. Among them, the key amino acids were Lys‐128, Lys‐161, Glu‐164, and Glu‐167, which significantly contributed to the binding energy (Figure S10D, Supporting Information).

Notably, the colocalization of MD2 with synapses significantly increased following IS (Figure 8F,G). MD2, also known as lymphocyte antigen 96, is a secreted protein widely expressed throughout the body. While MD2 is traditionally recognized as an auxiliary molecule that binds to TLR4, facilitating the specific recognition of lipopolysaccharides (LPS), recent research suggests that MD2 may have broader functional roles.^[^ ^29^, ^30^ ^]^ Thus, we hypothesized that Clec7a, a phagocytic receptor on the surface of microglia, plays a role in the phagocytosis of synapses by recognizing synaptic MD2.

To further clarify the roles of Clec7a and MD2 in microglial phagocytosis of synaptosomes, we examined the phagocytosis of synaptic material in a system that allows direct assessment of microglial Clec7a function. We used a previously established in vitro synaptosome assay to quantify the phagocytosis of pHrodo‐conjugated synaptosomes by cultured microglia isolated from *Clec7a^i∆MG^* and *Clec7a^fl/fl^* littermate mice (Figure 8H). When fed synaptosomes from *MD2^wt/wt^* mice, *Clec7a^i∆MG^* microglia phagocytosed fewer synaptosomes than *Clec7a^fl/fl^* microglia (Figure 8I). When *Clec7a^i∆MG^* and *Clec7a^fl/fl^* microglia were incubated with synaptosomes from *MD2^ko/ko^* mice, microglial phagocytosis of synapses was reduced, suggesting that MD2 is required for synaptic phagocytosis. Furthermore, *Clec7a^i∆MG^* microglia did not phagocytose more *MD2^ko/ko^* synaptosomes than *MD2^wt/wt^* synaptosomes, suggesting that Clec7a may be the primary microglial receptor for MD2. These data indicate that MD2 serves as a ligand of Clec7a on synaptosomes to promote microglial Clec7a‐mediated phagocytosis in vitro.

## 3 Discussion

Microglia‐mediated synaptic phagocytosis has been shown to be associated with synapse loss and dysfunction in a variety of neurological disorders, including autism, schizophrenia, depression, and AD.^[^ ^7^, ^17^, ^31^, ^32^, ^33^ ^]^ The loss of synapses and disruption of synaptic plasticity are significant contributors to neurological deficits and our study demonstrated that phagocytosis of excitatory synapses by microglia triggers long‐term neurological dysfunction after IS. In this study, we identified a functional role for Clec7a in microglial synaptic phagocytosis, which may have broader implications for understanding the neurological mechanisms underlying synaptic vulnerability in stroke and other neurological disorders characterized by synaptic dysfunction.

Microglia are immune cells that reside in CNS and are widely distributed throughout the brain and spinal cord.^[^ ^34^, ^35^, ^36^, ^37^ ^]^ One of the primary functions of microglia is to consistently monitor the CNS microenvironment and maintain brain homeostasis. Depletion of microglia in a model of cerebral hemorrhage reduces neurological deficits and edema by promoting blood‐brain barrier integrity.^[^ ^38^ ^]^ Microglial depletion treatment was also found to alleviate learning deficits and improve neurocognitive outcomes in mice postsepsis.^[^ ^10^ ^]^ However, aged (18–19‐month‐old) mice fed with PLX5622 chow diet for 21 days to deplete microglia exhibited exacerbated neurological deficits and an increased cerebral infarct volume 24 h post‐tMCAO.^[^ ^39^ ^]^ In our present study, young (8–10‐week‐old) mice were fed a PLX5622 chow diet for 14 days to deplete microglia, and showed improved infarct volume and neurological function. This may be due to differences in glial responses between young and aged mice, as well as the timing of PLX5622 feeding for microglial depletion. Additionally, another study using 8‐week‐old mice in which microglia were depleted with PLX5622 for 7 consecutive days also revealed an improvement in tMCAO infarct volume, which is consistent with our findings.^[^ ^19^ ^]^

Recent research has revealed that microglia and astrocytes can phagocytose synapses via two crucial molecules, namely, MEGF10 and MERTK, leading to synapse loss, destruction of neural circuits and ultimately causing neurological dysfunction.^[^ ^20^ ^]^ Notably, inhibition of microglia‐mediated phagocytosis significantly increased the density of synapses in the periphery of the injury site, reduced brain damage, and improved motor and cognitive functions in mice.^[^ ^20^ ^]^ In this study, we found that microglia exhibited significant activation after IS and had an augmented capacity for synaptic phagocytosis, contributing to the development of neurological impairment. Pharmacological depletion of microglia reduced the stroke infarct volume and improved neurological and cognitive functions after stroke. In addition, RNA‐seq of microglia from ischemic mice revealed significant upregulation of phagocytosis and DAM genes. Clec7a, a significant DAM gene, showed notably elevated following IS, indicating its potential role in the response to ischemic injury. Given the growing number of literature and the emerging significance of Clec7a in neurological disease,^[^ ^25^, ^40^ ^]^ it presents a compelling target for further investigation.

Clec7a is a pattern‐recognition receptor expressed by myeloid phagocytes, such as macrophages, dendritic cells, and neutrophils. It can recognize β‐glucans present in the cell walls of fungi and ultimately leads to the initiation of direct antimicrobial activities, including phagocytosis and ROS production.^[^ ^41^, ^42^, ^43^, ^44^, ^45^ ^]^ Clec7a expression in neutrophils and monocytes regulates the expression of Osm, a neuroprotective factor, which has been shown to be beneficial for treating autoimmune neuroinflammation.^[^ ^46^ ^]^ Recent research has demonstrated that Clec7a activation triggers the subsequent activation of SYK, leading to the transformation of microglia into DAM. This transformation enhances the clearance of Aβ plaques.^[^ ^25^, ^40^ ^]^ The activation of SYK by a Clec7a agonist directly restores the ability of microglia to clear Aβ plaques in TREM2^R47H^ mutant mice, an effect that was previously unattainable with SYK activation alone.^[^ ^40^ ^]^ In this study, we identified the involvement of Clec7a as a receptor for microglia‐mediated synapse engulfment in both IS mouse models and in vitro investigations. These findings are consistent with previous research on primary macrophages from Clec7a‐deficient mice, which showed that Clec7a inactivation reduces the ability of macrophages to phagocytose *C. albicans* cells,^[^ ^43^ ^]^ similar to our observations regarding microglia and synaptosomes. Furthermore, Clec7a expression was significantly lower in microglia from sham mice than in those from mice subjected to IS. Knockdown of microglial Clec7a markedly inhibited microglia‐mediated phagocytosis of synapses. Overall, the impact of Clec7a on microglial phagocytosis was confirmed in this study.

Microglia‐mediated synaptic phagocytosis requires the binding of surface receptors to ligands; for example, C1q and C3 localize to developing synapses and promote microglial engulfment of synaptic inputs through microglial CR3.^[^ ^24^, ^47^ ^]^ These findings indicate that immune signaling pathways within the CNS can regulate synaptic pruning. As previously confirmed, Clec7a is a receptor mediating microglial phagocytosis of synapses. Potential ligands for Clec7a include β glucan,^[^ ^25^, ^48^ ^]^ Lgals9,^[^ ^45^, ^46^ ^]^ annexins,^[^ ^49^ ^]^ and vimentin.^[^ ^50^, ^51^ ^]^ However, there were no significant differences in the colocalization of Lgals9, the most common ligand for Clec7a, with synapses before and after IS (Figure S11, Supporting Information).

Our prior research revealed a notable MD2 expression increase in neurons poststroke, indicating that MD2 might be located at synapses, but the role of MD2 in synapses has not been determined.^[^ ^29^ ^]^ Additionally, our research revealed no significant differences in the colocalization of MD2 with synaptic pruning signals (C3, C1q) or signals refractory to pruning (CD47) before and after IS (Figure S12, Supporting Information). Here, our findings not only indicate that MD2 is present in synapses but also provide evidence for an interaction between MD2 and Clec7a, suggesting that MD2 may serve as a ligand for Clec7a. This interaction may represent an intriguing mechanism for microglial Clec7a‐mediated phagocytosis of synapses. Based on the findings of our in vitro investigation, which demonstrated an interaction between residues 150–174 of the extracellular domain of Clec7a and residues 85–109 of MD2, we performed MD simulations, and the results of statistical analysis strongly supported the notion that Clec7a interacts with MD2. Current IP, LS/MS, MD simulation, and MST data provide evidence supporting the interaction between Clec7a and MD2, underscoring the significance of MD2 as a novel ligand for microglial Clec7a. Collectively, these findings indicate that targeting microglial Clec7a may be a promising therapeutic strategy for ameliorating neurodegeneration associated with synapse loss.

After an ischemic insult, the expression of Clec7a was strongly upregulated in microglia. Our experimental data showed that Clec7a is a key factor of ischemic stroke, indicating that therapeutic reduction of Clec7a function might be beneficial for the treatment of ischemic stroke. Further long‐term observations are required to determine whether Clec7a influences neuronal and synaptic functions through alternative pathways or ligands following IS. In addition, future efforts are required to determine whether modulating these pathways can restore functional or impaired synapses.

## 4 Experimental Section

### Mice

Male C57BL/6 mice were purchased from the Shanghai Model Organisms Center, Inc. *MD2^ko/ko^* mice were obtained from Cyagen Biosciences (S‐KO‐03021), *Clec7a^fl/fl^* mice were obtained from Gempharmatech (strain no. T009277), and *Cx3cr1^CreERT2^* mice were obtained from Jackson Labs (IMSR_JAX:02 1160). *Clec7a^fl/fl^* mice were crossed with *Cx3cr1^CreERT2^* mice to ensure constitutive microglial *Clec7a* deletion, these mice were named *Clec7a^i∆MG^*. To stimulate Cre‐ERT2 postnatally, 6‐week‐old male mice were intraperitoneally injected with 75 mg kg^−1^ tamoxifen (Sigma) in corn oil (Sigma) for 7 consecutive days. The mice were housed alongside littermates, provided with unlimited access to food and water, and maintained on a 12 h light/dark cycle. The ambient temperature of the room was maintained between 20 and 22 °C at 55% humidity. All procedures relating to the care of animals, including their upkeep, were sanctioned by the Ethics Committee of the Laboratory Animal Centre of Tongji University (Approval Number: TJBH2AC202334). The procedures were implemented in compliance with the Guide for the Care and Use of Laboratory Animals of Tongji University (Shanghai, China) and the International Guide for the Care and Use of Laboratory Animals. Mouse genotypes were determined through PCR using tail genomic DNA.

### Cell Culture

The BV2 mouse microglia cell lines was cultured in DMEM (Gibco, Thermo Fisher Scientific) supplemented with 10% fetal bovine serum (FBS; Gibco, Thermo Fisher Scientific). The cells were maintained at 37 °C in a humidified incubator with 5% CO_2_ and 95% air. BV2 cell lines were a gift from Dr. Jialin Charles Zheng's laboratory at Tongji University.

### Cell Transfection

For Clec7a knockdown, a negative control RNA sequence (siNC) or a small interfering RNA targeting Clec7a (ObiO Shanghai, China) was used with lipofectamine 2000 transfection reagent (Invitrogen). The primer sequences used was: GGGAAGAGCUGUUACCUAU, and the cells were used for further experiments after 48 h. For Clec7a overexpression, BV2 cells were plated into 6‐well plates (1 × 10^6^ cells per well) and incubated overnight. Subsequently, lentivirus (Shanghai GeneChem, China) and polybrene (CAS 28728‐55‐4, Santa Cruz, 5 µg ml^−1^) were added to each well. Transfection efficiency was assessed by green fluorescent protein (GFP) detection, and cells were selected with puromycin (CAS 58–58–2, Santa Cruz, 2 µg ml^−1^). Stable cell lines were established after 1 week of selection. The expression of Clec7a mRNA was examined by qRT‐PCR. For generation of microglial primary cultures, P2–3 tamoxifen‐induced *Clec7a^i∆MG^* or *Clec7a^fl/fl^* mice were decapitated, the brain was dissected from the skull, and the meninges were removed in ice‐cold HBSS supplemented with 5% FBS. After a 30 min incubation at 37 °C in 0.25% trypsin (25 300 054, Thermo Fisher Scientific), the cells were centrifuged, washed twice with 1 × HBSS, and resuspended in complete medium (DMEM, 10% FBS, 100 U ml^−1^ penicillin, and 100 mg ml^−1^ streptomycin). The cell suspension was evenly distributed into three poly‐D‐lysine (P6407, Sigma‐Aldrich) coated T‐25 flasks. Half of the medium was replaced after 3 days and subsequently changed every other day. Between days 10 and 14 postplating, the media were aspirated from each flask, and the cells were used.

### Transient Middle Cerebral Artery Occlusion (tMCAO) Model

Adult male mice (8‐10 weeks old, 22–25 g) were subjected to transient focal cerebral ischemia induced by intraluminal occlusion of the left middle cerebral artery for 1 h as previously described.^[^ ^52^ ^]^ Sham‐operated animals were anesthesia, and the arteries were exposured without MCA occlusion. Briefly, mice were anesthetized with 1.5% isoflurane in 67%:30% N_2_O/O_2_ under spontaneous breathing for anesthesia maintenance. A monofilament with a silicon‐coated tip was introduced into the common carotid artery, advanced to the origin of the MCA, and left in place to limit MCA blood flow for 1 h. The rectal temperature was controlled at 37.0 ± 0.5 °C using a temperature‐regulated heating pad during surgery. Regional cerebral blood flow (CBF) was measured in all stroke animals using laser speckle contrast imaging (RWD Life Science Co., Ltd.). Animals that did not display at least 70% reduction in regional CBF during tMCAO compared to preischemic animals were excluded from further experimentation. The surgeries and quantification were performed by investigators who were blinded to the animal genotypes and experimental groups.

### 2,3,5‐Triphenyltetrazolium Chloride (TTC) Staining

Mice were euthanized, and their brains were removed and frozen. Coronal slices were made into six 1‐mm‐thick sections (rostral to caudal) using a mice brain slicer, the slices were later subjected to 30 min incubation in 0.2% TTC at 37 °C and fixed in 4% PFA. Data were collected for each mouse from 5 brain sections stained with TTC. ImageJ (ver2.14.0, NIH) was employed for analyzing. The infarction area was defined as the unstained region of the fixed brain sections, with the margins being manually outlined. Infarct volume was determined using the subsequent formula to adjust for bias stemming from brain edema effects: percentage of infarct = (LT‐RN) × 100/RT, where LT signifies the overall volume of the left hemisphere, RN indicates the volume of the non‐infarcted area in the right hemisphere, and RT is the complete volume of the right hemisphere.

### Magnetic Resonance Imaging (MRI)

MRI were conducted by using an 11.7‐T horizontal system (Bruker Avance). Evaluation of infarct volume at 1‐day post‐tMCAO utilized diffusion‐ or T2‐weighted images. Mice were anesthetized with 4% isoflurane and maintained at 1% isoflurane in ambient air. They were positioned in stereotactic holders with low‐temperature, helium‐cooled, closed‐loop RF coils. Scout images included three perpendicular brain slices. T2‐weighted axial slices (0.3 mm thickness, 45 slices total) were acquired using a fast spin‐echo sequence (FOV = 100 mm, in‐phase resolution 256 µm, TE = 26 ms, TR = 3000 ms, echo train length = 8, NA = 2). Subsequently, diffusion‐weighted images (DWI) were obtained with an Echo Planar Imaging (EPI) sequence (FOV = 100 mm, b values = 0/1000 mT/m, TE/TR = 20/3500 ms, echo train length = 23, in‐phase resolution 92 µm, NA = 5). Lesion extent calculations used these images, and infarct volume was quantified using Itksnap software (ver 4.0.2).

### mNSS Score

Table S1 (Supporting Information) displays the mNSS, which comprises motor, sensory, reflex, and balance assessments. The neurological function was rated on a scale of 0 to 18, with 0 being the normal score and 18 indicating maximal deficit. The injury severity scores ranged from 1 to 6 for mild damage, from 7 to 12 for moderate damage, and from 13 to 18 for severe impairment.

### Rotarod Test

The experiment entailed performing the rotarod test using the Rotarod device (Panlab, LE8505), in which the speed was gradually accelerated from 5 to 40 rpm over a period of 5 min. The duration of time before the mice fell off the rotarod was measured, and the data collected were expressed as mean values from three independent trials. Mice underwent pre‐training one day before tMCAO, and the recorded data were labeled as preoperative (pre). Following this, repetition testing took place at 1, 3, 7, 14, 21, and 28d post tMCAO.

### Adhesive Removal Test

The experiment involved conducting the adhesive removal test using 2 × 3 mm tape. Adhesive tapes were placed on the ipsilateral or contralateral forepaw of the mouse to assess sensory and motor function after tMCAO. The duration taken by the mouse to touch and remove the tape was measured up to 90 s, at which time the timer was halted. The outcome data was expressed as a mean value obtained from three trials. The preoperative data (pre) was obtained by recording measurements from one day prior to tMCAO. Repeat assessment using the adhesive removal test was conducted at 1, 3, 7, 14, 21, and 28d post tMCAO.

### Morris Water Maze

Morris water maze test was conducted in a black pool with a diameter of 150 cm and depth of 60 cm. The height of the platform was 40 cm, and water (20 °C) dyed white with titanium dioxide was added to just submerge the platform (such that the platform was maintained at 1 cm below the water surface). The tank was divided into four equal quadrants (SE, SW, NW, NE) and spatial clues were placed at the borders of each quadrant on the pool wall above the water. The platform was fixed in the middle of SE until the end of the test. Before testing, the experimental mice were placed in the behavioral testing room for 30 min for habituation. At d22, the subject mice were trained for 5 consecutive days (4 trials per day). The experimental mouse was placed in a random starting position facing the pool well. After the mouse found the platform, it stayed on the platform for 10 s. The time for each mouse to find the platform was defined as escape latency. A video tracking system recorded the swimming motions of the animals, and the data were analyzed using motion‐detection software for the MWM. At the end of the reference training (d27), the platform was removed from the pool, and the mouse was placed in the NW quadrant. Each mouse was allowed to swim for 60 s. Time in each quadrant, number of entries into each quadrant, and number of crossings over the previous site of the platform were recorded.

### Western Blot (WB) Analysis

The frozen ischemic penumbra tissue was transferred to a Triton‐based lysis buffer containing protease inhibitors and homogenize using JY92‐IIN (Scientz). Protein samples were load and separated by SDS‐PAGE on a 12.5% tris‐glycine gel. After blocking with 5% milk, the membrane incubated with the following primary antibodies: rabbit anti‐Clec7a (1:500, mabg‐mdct, Invivogen), mouse anti‐MD2 (1:500, 11784‐1‐AP, Proteintech), rabbit anti‐Tubulin (1:3000, #AF7011, Affinity), and their respective HRP‐conjugated secondary antibodies (BioRad). Then transfer to a nitrocellulose membrane and probe with the relevant antibodies in 5% milk. Perform densitometric analysis of the bands using ImageJ software.

### Immunofluorescence Staining

Mice brain or cells were first fixed with 4% PFA for 24 h. The fixed tissue was then embedded in OCT and sectioned into 20‐µm sections. These sections were sealed with 10% goat serum for 60 min at room temperature. After blocking, sections were rinsed and incubated with the following primary antibodies in dilution buffer overnight at 4 °C: anti‐Iba‐1 (1:500, 019–19741, WAKO); anti‐CD68 (1:500, 14‐0681‐82, Invitrogen); anti‐PSD95 (1:100, MA1‐046, Invitrogen); anti‐Synaptophysin (1:100, S5768, Sigma); anti‐Clec7a (1:100, mabg‐mdct, Invivogen); anti‐MD2 (1:100, GTX85517, GTX85121, GeneTex), anti‐C1q (1:100, ab11861, abcam), anti‐C3 (1:100, ab11862, abcam), anti‐Lgals9 (1:100, abs143512, Absin). Afterward, the sections were incubated with DAPI and the corresponding secondary antibodies (all from Abcam and diluted 1:500) to visualize the primary antibodies: goat anti‐rabbit Alexa Fluor 488 (ab150077); goat anti‐rabbit Alexa Fluor 594 (ab150080); goat anti‐mouse Alexa Fluor 488 (ab150113); goat anti‐mouse Alexa Fluor 647 (ab150115); goat anti‐rat Alexa Fluor 647 (ab150167). Sections were mounted and imaged using an Olympus FV3000 confocal microscope and FV31S‐SW software.

### 3D Reconstruction

The 20 µm brain sections were stained with anti‐Iba‐1, anti‐CD68, and anti PSD95 or anti‐Synaptophysin overnight at 4 °C, followed by fluorochrome‐conjugated secondary antibody staining. An Olympus FV3000 confocal microscope with a × 60 oil objective was utilized to perform confocal images. The microglia were scanned from top to bottom with 0.22‐µm steps in the z direction with 2048 × 2048‐pixel resolution. The images were then analyzed blindly using Imaris 9.0 software. To determine branch length and the number of processes, the “Filaments” function of Imaris 9.0 software was employed. Microglial engulfment was evaluated with Imaris 9.0 software for the creation of a 3D surface rendering of the microglia, and the module of Surface in Imaris 9.0 software measured the microglia's surface area. The findings were utilized for 3D reconstruction and morphological examination of microglia.

### Stereotactic Intracerebral Injection

Four to six weeks old WT C57BL/6 mice were anesthetized by 2% isoflurane. AAV (0.5 µl; 9 × 10^12^ viral genomes per mL) was then injected into 2 mm lateral to the bregma and 3.5 mm under the dura. The microinjections were performed at a rate of 0.05 µL min^−1^ for 10 min by an automated stereotactic injection apparatus (78‐8130, KD Scientific). AAV‐hSyn‐Synaptophysin‐mcherry‐EGFP, AAV‐GAD67‐Synaptophysin‐mCherry‐EGFP, AAV‐hSyn‐PSD95‐mcherry‐EGFP, and AAV‐hSyn‐Gephyrin‐mcherry‐ EGFP were bilaterally injected using a glass electrode with a 10‐µL micro‐syringe (Hamilton, Bonaduz). Following the lentivirus injection, the needle was left in place for an additional 10 min after each injection.

### Pharmacological Depletion of Microglia

Oral CSF1R inhibitor PLX5622 (D20010801, SYSE Biotechnology) was added to the AIN‐76A standard food at a concentration of 1200 parts per million. Five to six weeks old mice were fed PLX5622 or AIN‐76A food without PLX5622, starting 14 days before surgery and continues until the end of the experiment.

### Isolation of Microglia from Ischemic Stroke Mice Brain

Eight weeks old mice were deeply anesthetized with isoflurane and transcranial perfused with ice‐cold saline. The infarcted brain was immediately removed and washed with pre‐cooled phosphate‐buffered saline (PBS). Brain tissue was dissociated mechanically and enzymatically using the Neural Tissue Dissociation Kit (130‐093‐231, Miltenyi Biotec) according to the manufacturer's instructions. After removal of myelin by centrifugation using a percoll (17 089 109, Cytiva) gradient, total cell sediment was resuspended with 5% MACS buffer (130‐093‐376, Miltenyi Biotec) and microglia were isolated by anti‐CD11b‐coated MicroBeads (130‐093‐634, Miltenyi Biotec). For RNA‐seq experiments, isolated microglia were collected from 30 mice per sample. For RNA extraction, isolated microglia were collected from 8–10 mice per sample.

### Bulk RNA‐Seq and Analysis

The microglia were isolated at 7 days following ischemic stroke, each RNA sample was pooled from 30 mice. Total RNA was extracted using Trizol reagent, RNA purity was tested using the Nano Photometer spectrophotometer (IMPLEN, Westlake Village). A total of 1 µg total RNA from each sample (*n* = 3 per group) was used for library construction according to the manufacturer's protocols (SQK‐PCS109). Briefly, the reverse transcriptase was used to enrich full‐length cDNAs and added defined PCR adapters to both ends of the first strand of cDNA and following cDNA PCR for 14 circles with LongAmp Tag DNA polymerase (New England Biolabs, Ipswich, MA, USA) (8 min for elongation time). The PCR products were then subjected to ONT adaptor ligation using T4 DNA ligase (New England Biolabs, Ipswich, MA, USA). Agencourt XP beads (Beckman Coulter, Brea, USA) were used for DNA purification. The final cDNA libraries were subjected to FLO‐MIN109 flow cells and analyzed on a PromethION platform at Biomarker Technology Company (Beijing, China). Interactions between microglia phagocytosis‐related genes were demonstrated according to the STRING database (v11.0).

### Quantative Real‐Time PCR (qRT‐PCR)

Total RNA was isolated using TRIzol regent (R411‐01, Vazyme) according to the manufacturer's instructions. Next, RNA purity and concentration were assessed by Nanodrop. mRNA was converted to cDNA using the 5 × All‐in‐on qRT SuperMix reverse transcription kit as described by the manufacturer (R333, Vazyme). For qRT‐PCR, 40 ng of cDNA was loaded in triplicates per gene in a total volume of 20 µl using the SYBR green PCR master mix as described by the manufacturer (Q712‐02, Vazyme). Reactions were performed using a real‐time PCR system (QuantStudio 7 Flex, Thermo Fisher Scientific). The three Ct values were averaged, and the data were displayed as the geometric mean of the housekeeping gene (GAPDH) using the Ct delta method (2^‐ΔΔCt^). Sequences of the primers can be found in Table S2 (Supporting Information).

### Isolation of Synaptosomes and Conjugation to pHrodo

Eight weeks old mice were pooled per experiment. In brief, mice were intracardiac perfused with 10 ml ice‐cold PBS. The brain was homogenized, and the homogenate (synaptosomes) was suspended in Syn‐Per Synaptic Protein Extraction Reagent (87 793, Thermo Fisher Scientific). Then centrifuged at 1200 g for 10 min at 4 °C and the supernatant was saved as total homogenate fraction (THF). The THF was centrifuged again at 15 000 g for 20 min at 4 °C and supernatant was saved. Five microliters synaptosomes and 0.5 µl pHrodo red (P35362, Invitrogen) were added per 20 000 cells, and the mixture was incubated for 45 min away from light, washed twice with PBS, centrifuged to discard the supernatant, and resuspended in 200 µl of medium, and 1:100 was added to the cultured cells.

### Co‐Immunoprecipitation (Co‐IP)

Co‐IP was used to detect whether there was a direct interaction of Clec7a and MD2. Mouse MD2 was immunoprecipitated from 400–500 µg of protein per sample using 1.5 µg of mouse MD2 biotinylated antibody (GTX85121, GeneTex), and Mouse Clec7a was immunoprecipitated from 400–500 µg of protein per sample using 1.5 µg of mouse Clec7a biotinylated antibody (GTX41467, GeneTex). After overnight incubation at 4 °C, biotinylated antibody was pulled down using NeutrAvidin agarose beads (26 149, Thermo Fisher Scientific) for at least 2 h at 4 °C. Avidin beads were collected by centrifugation at 2000 g for 3 min, washed four times with IP lysis buffer and boiled in SDS–PAGE loading buffer for immunoblotting. Blots were probed with rabbit anti‐Clec7a (1:500, mabg‐mdct, Invivogen) and mouse anti‐MD2 (1:500, 11784‐1‐AP, Proteintech).

### Molecular Dynamics (MD) Simulations

In this study ZDOCK 3.0.21 was used to predict the binding mode of Clec7a and MD2. Prior to the start of docking, the structure files of each of these proteins were obtained from the PDB. Subsequently, they were processed using PyMol 2.5.5,^[^ ^53^ ^]^ including removal of water molecules, removal of hydrogen atoms, and non‐target structural proteins. Docking studies were performed with the default configuration of ZDOCK 3.0.2 for global rigid docking. After completion of docking energy minimization was performed using AMBER18 under ff14SB force field. Finally, the protein complex conformation after energy minimization was evaluated for binding energy using the online tool prodigy (https://wenmr.science.uu.nl/prodigy/). Binding modes based on the optimal binding energies were visualized and analyzed using PyMOL 2.5.3, as well as for subsequent molecular dynamics simulation studies. All‐atom molecular dynamics simulations based on the small molecule and protein complexes obtained by the above docking as initial structures were performed separately, and the simulations were carried out using the AMBER 18 software.^[^ ^54^ ^]^ Prior to the simulations, the proteins were described using the ff14SB protein force field.^[^ ^55^ ^]^ The LEaP module was used for each system to add hydrogen atoms to the system, a truncated octahedral TIP3P solvent box was added at 10 Å from the system^[^ ^56^ ^]^ and Na^+^/Cl^‐^ was added to the system for balancing the system charge, and finally the topology and parameter files used for the simulations were output. Molecular dynamics simulations were performed using AMBER 18 software.^[^ ^57^ ^]^ Prior to the simulation, the system was subjected to energy optimization, including the steepest descent method with 2500 steps and the conjugate gradient method with 2500 steps. After the system energy optimization was completed, a 200 ps warming of the system at a fixed volume and constant rate of warming was used to slowly increase the temperature of the system from 0 to 298.15 K. An NVT (isothermal isobaric) tethered simulation was carried out for 500 ps to further distribute the solvent molecules homogeneously in the solvent box at a maintenance temperature of the system at 298.15 K. The system temperature was then adjusted to the temperature of the solvent box by using the NPT (isothermal isobaric) system simulation. Finally, the NPT (isothermal isobaric) case was performed for 500 ps of equilibrium simulation for the whole system. Finally, NPT (isothermal isobaric) tethered simulations of 100 ns were performed for each of the two composite systems under periodic boundedness conditions. For the simulations, the non‐bond truncation distance was set to 10 Å. The Particle mesh Ewald (PME) method was used to calculate long‐range electrostatic interactions,^[^ ^58^ ^]^ the SHAKE method was used to limit the bond lengths of the hydrogen atoms,^[^ ^59^ ^]^ and Langevin's algorithm was used for temperature control,^[^ ^60^ ^]^ in which the collision frequency γ was set to 2 ps‐1. The system pressure was 1 atm, and the integration step was 2 fs, with trajectories saved for subsequent analyses at 10 ps intervals. The trajectories were saved for subsequent analyses.

### LC‐MS/MS

Both LC–MS/MS and affinity purification coupled with MS (AP‐MS) interactome analysis were carried out. tMCAO mouse brains were lysed in the lysis buffer of 50 mm Tris‐HCl, protease inhibitor cocktail, phosphatase inhibitor and 0.002% Zwittergent and kept on ice for an additional 30 min. After centrifugation, two aliquots of the supernatant were incubated with anti‐mouse Clec7a and anti‐mouse MD2 on a rotator, respectively. After three washes, immunoprecipitated proteins were used for further LC–MS/MS analysis. Protein was digested with trypsin. The extracted tryptic peptides were dried in a speedVac, analyzed by nano‐electrospray ionization (nanoESI)‐LC–MS/MS with an Orbitrap Exploris 480 mass spectrometer coupled to a dionex nano‐LC system with a 75 cm long analytical column packed with C18 particles (Acclaim, 5 µm). Buffer A was 0.1% Formic acid (FA) aqueous solution, Buffer B is 0.1% FA in Acetonitrile (ACN). The flow rate of the mobile phases was 300 nL min^−1^ with the following gradient: 0–12 min 9% B, linearly up to 40% in 188 min, linearly up to 95% in 10 min, kept at 95% for 5 min, linearly down to 9% in 5 min, and finally kept at 9% for 5 min for column equilibration. MS spectra were acquired in the data‐dependent mode with m/z range 350–1700, mass resolution 120 k (m/z 200), automatic gain control (AGC) target 3e6, and maximal ion injection time 50 ms. MS/MS spectra was acquired for the Top15 ions with the settings of mass resolution 30 k, AGC target 5e5, maximal ion injection time 120 ms, normalized collision energy (NCE) 32%, isolation window 1.4 m/z, and dynamic exclusion 30 s. The acquired LC‐MS/MS raw were searched using Proteome Discoverer (ThermoFisher Scientific) against a UniProt database (Clec7a, PDB Code: 2BPD; MD2, PDB Code: Q9JHF9)) for peptide and protein identification. The search parameters included a maximum of two missed cleavages, carbamidomethylation at cysteine as a fixed modification and N‐terminal acetylation, deamidation at asparagine and glutamine and oxidation at methionine as variable modifications. The mass tolerance for both precursor and fragment ions were 10 ppm. The maximum modifications per peptide was set to 5, and the maximum charge state was set at 7. The target–decoy analysis was chosen for false discovery rate (FDR) control of 1%. The minimum peptide length was set to 5.

### Microscale Thermophoresis (MST)

MST was conducted using Monolith NT. 115 (NanoTemper, German) at 20 °C. The purified human Clec7a protein (Cat. 1859‐DC‐050) and human MD2 protein (Cat. 1787‐MD) were purchased from R&D Systems (USA). A standard buffer containing 20 mm HEPES (pH 8.0) and 150 mm NaCl was employed for protein solutions. The MST experiments were performed strictly following the User Manul. The evaluation of microscale thermophoresis data and quantification of Kd were conducted using NT Analysis Software (NanoTemper, German).

### Fluoro‐Jade C (FJC) Staining

FJC staining was performed using a commercial detection kit (Biosensis) to evaluate degenerating neurons as previously described [Systemic exosomal miR‐193b‐3p delivery attenuates neuroinflammation in early brain injury after subarachnoid hemorrhage in mice]. Data were presented showing the average number of FJC^+^ cells mm^−2^. The stained cells were quantified using ImageJ software.

### Brain Slice Electrophysiology

Adult (6‐8 weeks old) *Clec7a^fl/fl^* and *Clec7a^i∆MG^* mice were subjected to tMCAO and sham operation. 7 days later, they were anesthetized with tribromoethanol (100 mg kg^−1^, i.p.) and transcardially perfused with ice‐cold oxygenated (95% O_2_, 5% CO_2_) NMDG ACSF solution that included 93 mm NMDG, 93 mm HCl, 2.5 mm KCl, 1.25 mm NaH_2_PO_4_, 10 mm MgSO_4_·7H_2_O, 30 mm NaHCO_3_, 25 mm glucose, 20 mm HEPES, 5 mm sodium ascorbate, 3 mm sodium pyruvate, and 2 mm thiourea. After perfusion, the brain was quickly dissected out and immediately transferred into an ice‐cold oxygenated NMDG ACSF solution. Then the coronal plane of brain tissue was sectioned at a 300 µm thickness in the same buffer using a vibratome (VT1200 S, Leica, Germany). The brain slices containing the cortex were incubated in oxygenated NMDG ACSF at 32 °C for 10–15 min, then transferred to a normal oxygenated solution of ACSF (126 mm NaCl, 2.5 mm KCl, 1.25 mm NaH_2_PO_4_, 2 mm MgSO_4_·7H_2_O, 10 mm glucose, 26 mm NaHCO_3_, 2 mm CaCl_2_) at room temperature for 1 h. All chemicals used in slice preparation were purchased from Sigma‐Aldrich (St. Louis, MO, USA).

Slices were transferred to the recording chamber that was submerged and superfused with artificial cerebrospinal fluid at a rate of 2–3 mL min^−1^ at 28 °C. Whole‐cell patch‐clamp recordings were made from cortex neurons visualized with an Olympus BX61W1 microscope (equipped with mCherry filters) using infrared video microscopy and differential interference contrast optics. The recording pipettes (3–4 MΩ) were pulled with a micropipette puller (P2000, Sutter Instrument; USA) from borosilicate glass capillaries. For whole‐cell recording, the pipettes were filled with an internal solution containing 133 mm potassium gluconate, 18 mm NaCl, 0.6 mm EGTA, 10 mm HEPES, 0.3 mm Na_3_·GTP, 2 mm Mg·ATP (280 mOsm; pH 7.2). For action potentials evoked by current injections, a current‐step protocol (from 0 to +360 pA, with 20 pA increment for recording and the step currents during recording of action potential is 400 ms) was run and repeated. The PPR was measured by pairing electrical stimuli onto the cortex region with inter‐stimulus intervals of 50 ms and calculated by dividing the peak currents of the second postsynaptic response by the first one.

Electrophysiological recordings were acquired with a MultiClamp 700B amplifier (Molecular Devices) and Clampex 10.6 software. Signals were low‐pass filtered at 2 kHz and digitized at 10 kHz using Digidata 1550B (Molecular Devices). Recordings with Rs >30 MΩ were excluded from statistical analysis. Offline electrophysiological data analysis was performed with Clampfit 10.6 (Molecular Devices).

### Statistical Analysis

Graph Pad Prism 9 software was used as the statistics tool to determine whether there were statistically significant differences between groups. When comparing two conditions, two‐tailed unpaired Student's *t*‐test was used. When comparing more than two conditions, one‐way or two‐way analysis of variance (ANOVA) was performed, followed by Tukey's multiple comparisons test. Data were represented as mean ± SD, with a considered statistically significant *p* < 0.05.

## Conflict of Interest

The authors declare no conflict of interest.

## Supporting information

Supporting Information

Supplemental Table 4

Supplemental Table 7

## Data Availability

The data that support the findings of this study are available from the corresponding author upon reasonable request.
