# Supplementary material for: Clec7a Worsens Long‐Term Outcomes after Ischemic Stroke by Aggravating Microglia‐Mediated Synapse Elimination
Source: Adv Sci (Weinh). 2024 Aug 1;11(36):2403064. doi: 10.1002/advs.202403064 (PMC11423142; doi:10.1002/advs.202403064)
Supplement: Supplementary file 1 — Supporting Information [file ADVS-11-2403064-s002.docx]

Supporting Information

Clec7a Worsens Long-Term Outcomes after Ischemic Stroke by Aggravating Microglia-Mediated Synapse Elimination

*Hanxi Wan^1, *^, Mengfan He^1, *^, Chun Cheng^1, *^, Kexin Yang^2, *^, Huanghui Wu^1^, Peilin Cong^1^, Xinwei Huang^1^, Qian Zhang^1^, Yufei Shi^1^, Ji Hu^2, #^, Li Tian^1, #^, Lize Xiong^1, #^*

Supplementary Table S1. Modified Neurological Severity Score points.

| Modified Neurological Severity Score points | | |
| --- | --- | --- |
| Motor tests | | 3 |
|  | Flexion of forelimb | 1 |
|  | Flexion of hindlimb | 1 |
|  | Head move >10° to vertical axis within 30 s | 1 |
| Placing rat on floor | | 3 |
|  | Normal walk | 0 |
|  | Inability to walk straight | 1 |
|  | Circling toward paretic side | 2 |
|  | Falls to paretic side | 3 |
| Beam balance test | | 6 |
|  | Balances with steady posture | 0 |
|  | Grasps side of beam | 1 |
|  | Hugs beam and 1 limb down from beam | 2 |
|  | Hugs beam and 2 limbs fall from beam, or spins on beam (>60 s) | 3 |
|  | Attempts to balance on beam but falls off (>40 s) | 4 |
|  | Attempts to balance on beam but falls off (>20 s) | 5 |
|  | Falls off, no attempt to balance or hang on to beam (<20 s) | 6 |
| Sensory tests | | 2 |
|  | Placing test (visual and tactile test) | 1 |
|  | Proprioceptive test (deep sensation, pushing paw against table edge to stimulate limb muscles) | 1 |
| Reflex absence and abnormal movements | | 4 |
|  | Pinna reflex (head shake when auditory meatus is touched) | 1 |
|  | Corneal reflex (eye blink when cornea is lightly touched with cotton) | 1 |
|  | Startle reflex (motor response to a brief noise from snapping a clipboard paper) | 1 |
|  | Seizures, myoclonus, myodystonia | 1 |
| Maximum points | | 18 |

Supplementary Table S2. Real-time PCR primers

| Gene |  | Primer |
| --- | --- | --- |
| *GAPDH* | F | AGGTCGGTGTGAACGGATTTG |
|  | R | TGTAGACCATGTAGTTGAGGTCA |
| *Clec7a* | F | GACTTCAGCACTCAAGACATCC |
|  | R | TTGTGTCGCCAAAATGCTAGG |
| *Abca1* | F | GCTTGTTGGCCTCAGTTAAGG |
|  | R | GTAGCTCAGGCGTACAGAGAT |
| *Anxa2* | F | ATGTCTACTGTCCACGAAATCCT |
|  | R | CGAAGTTGGTGTAGGGTTTGACT |
| *Apoe* | F | CTGACAGGATGCCTAGCCG |
|  | R | CGCAGGTAATCCCAGAAGC |
| *Axl* | F | ATGGCCGACATTGCCAGTG |
|  | R | CGGTAGTAATCCCCGTTGTAGA |
| *C3* | F | CCAGCTCCCCATTAGCTCTG |
|  | R | GCACTTGCCTCTTTAGGAAGTC |
| *Cd63* | F | GAAGCAGGCCATTACCCATGA |
|  | R | TGACTTCACCTGGTCTCTAAACA |
| *Cxcl16* | F | CCTTGTCTCTTGCGTTCTTCC |
|  | R | TCCAAAGTACCCTGCGGTATC |
| *Tnf* | F | CCCTCACACTCAGATCATCTTCT |
|  | R | GCTACGACGTGGGCTACAG |
| *Il1b* | F | GCAACTGTTCCTGAACTCAACT |
|  | R | ATCTTTTGGGGTCCGTCAACT |
| *Itgb2* | F | CAGGAATGCACCAAGTACAAAGT |
|  | R | CCTGGTCCAGTGAAGTTCAGC |
| *Lgals3* | F | AGACAGCTTTTCGCTTAACGA |
|  | R | GGGTAGGCACTAGGAGGAGC |
| *Siglec* | F | CAGGGCATCCTCGACTGTC |
|  | R | GGAGCATCGTGAAGTTGGTTG |
| *Tlr2* | F | GCAAACGCTGTTCTGCTCAG |
|  | R | AGGCGTCTCCCTCTATTGTATT |

Supplementary Table S3. Microscale thermophoresis (MST) results

| Target Name: | Clec7a |
| --- | --- |
| Target Concentration: | 50 nM |
| Ligand Name: | MD2 |
| Ligand Concentration: | 10 μM to 0.00122 μM |
| n: | 3 |
| Excitation Power: | 60% |
| MST Power: | 40% |
| Temperature: | 25.0 °C |
| Kd: | 2.9642E-06 |
| Kd Confidence: | ± 1.1113E-06 |
| Response Amplitude: | 13.156956 |
| TargetConc: | 5E-08 [Fixed] |
| Unbound: | 887.61 |
| Bound: | 874.45 |
| Std. Error of Regression: | 0.84312135 |
| Reduced X^2^: | 0.50872181 |
| Signal to Noise: | 17.094482 |


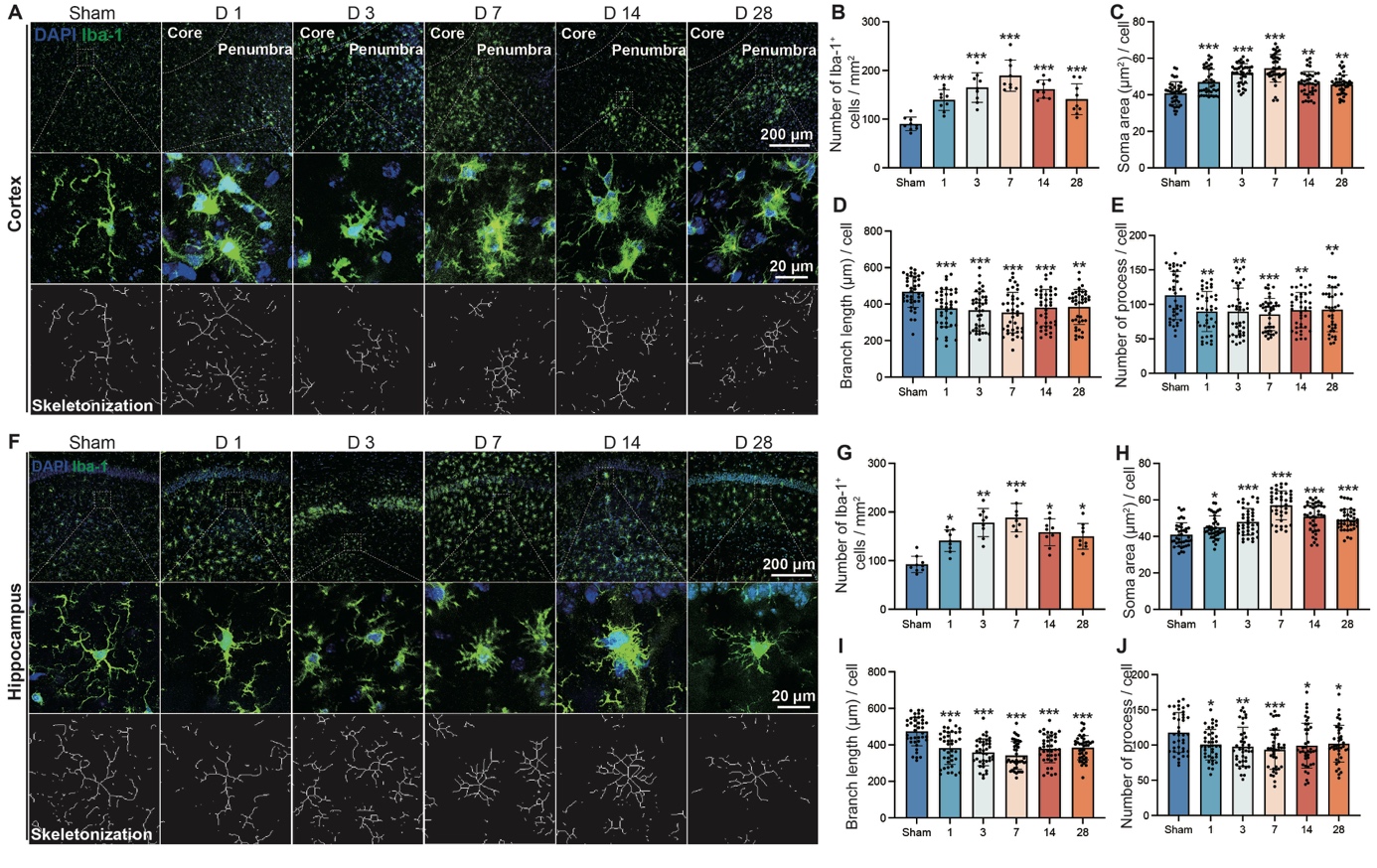


**Figure S1.** **Microglial activation in the cortex and hippocampus after ischemic stroke.** A. Representative immunohistochemical images (upper panels), magnified images (middle panels), and skeletonized images (lower panels) of Iba-1+ microglia in the cortex of sham mice and at 1, 3, 7, 14, and 28 days after tMCAO. Scale bars, 200 μm. B-E. Analysis and quantification of microglial morphological parameters, including the number of cells (B), the soma area (C) of the projections of Iba-1+ cell bodies (each symbol indicates one cell), the branch length (D) and the number of processes (E) per cell in the cortex of sham mice and at 1, 3, 7, 14, and 28 days after tMCAO. F. Representative immunohistochemical images (upper panels), magnified images (middle panels), and skeletonized images (lower panels) of Iba-1+ microglia in the hippocampus of sham mice and at 1, 3, 7, 14, and 28 days after tMCAO. Scale bars, 200 μm. G-J. Analysis and quantification of microglial morphological parameters, including the number of cells (G), the soma area (H) of the projections of Iba-1+ cell bodies (each symbol indicates one cell), the branch length (I) and the number of processes (J) per cell in the cortex of sham mice and tMCAO mice. Statistics are derived from 18 slices, n = 6 mice for each group. In B-E and G-J, significance was calculated using one-way ANOVA, Tukey’s multiple comparisons test. Data are presented as mean ± SD. **p* < 0.05, ***p* < 0.01 and ****p* < 0.001 *vs.* Sham.


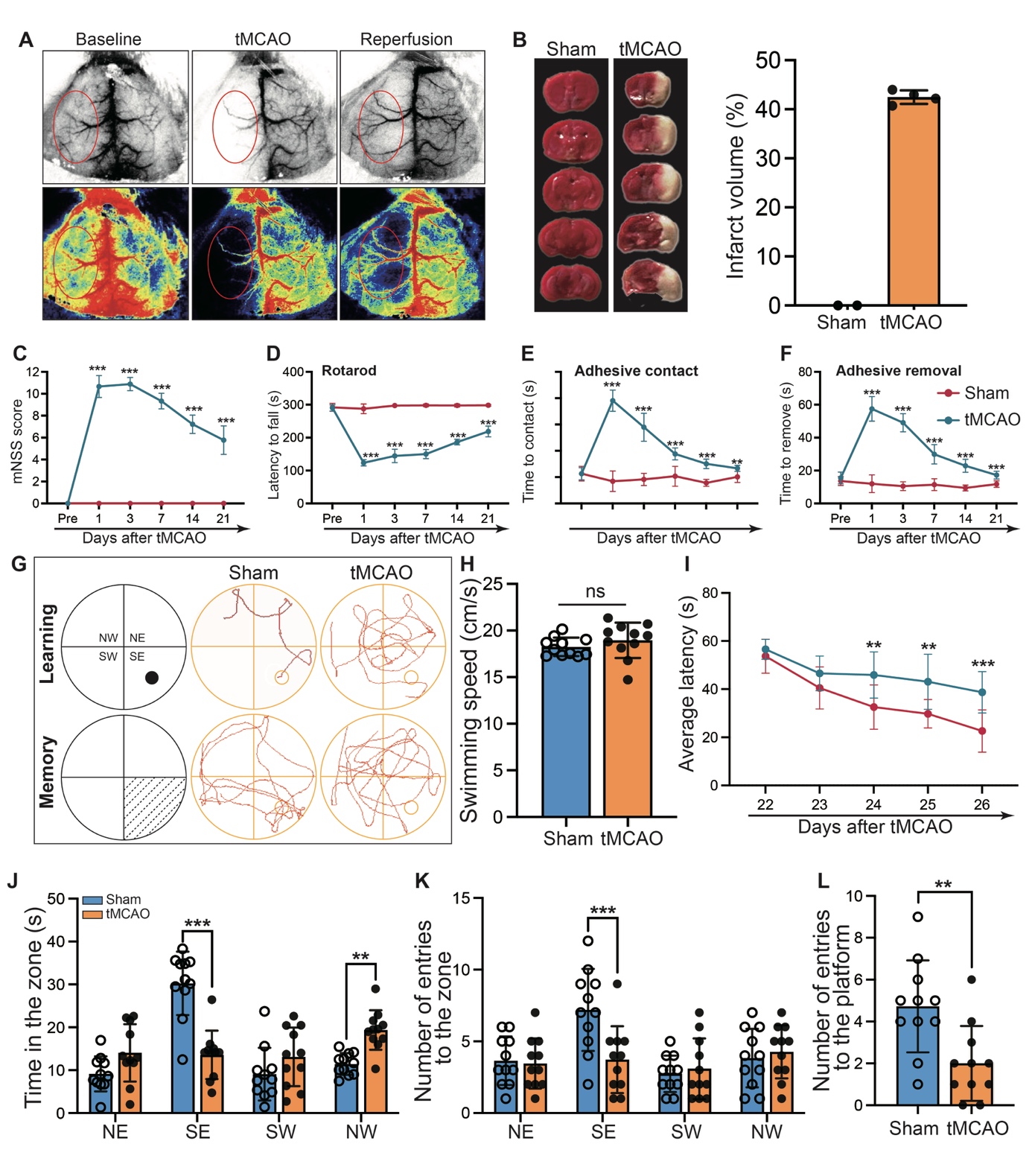


**Figure S2.** **Long-erm Neurological dysfunction and cognitive impairment after ischemic stroke.** A. Cerebral blood flow (CBF) was monitored at baseline, during tMCAO and 40 min following reperfusion using a 2-dimensional laser speckle imaging system. B. Representative images of TTC-stained coronal brain sections (left) from mice after tMCAO. The right panel shows the quantification of the relative infarct volume. n = 4 mice per group. C-F. Long-term neurological dysfunction after tMCAO was assessed with the mNSS (C), rotarod test (D), adhesive contact test (E), and adhesive removal test (F). n=8 mice per group. G-L. Cognitive deficits were evaluated 22 to 27 days after tMCAO via the Morris water maze test. The panels show a schematic diagram and representative navigation trajectories for learning and memory (G), swim speeds (reflecting gross locomotor functions) (H), summarized escape latency data (reflecting spatial learning) (I), time spent in the four quadrants (reflecting spatial memory) (J), the number of entries into the four quadrants (reflecting spatial memory) (K), and the number of entries into the platform region (reflecting spatial memory) (L). n=11 mice per group. In B, H and L, significance was calculated using two-tailed unpaired Student’s *t* test, in C-F and I-K, significance was calculated using two-way ANOVA, Tukey’s multiple comparisons test. The data are presented as mean ± SD. ***p* < 0.01 and ****p* < 0.001, ns indicates no significant difference. tMCAO: transient middle cerebral artery occlusion.


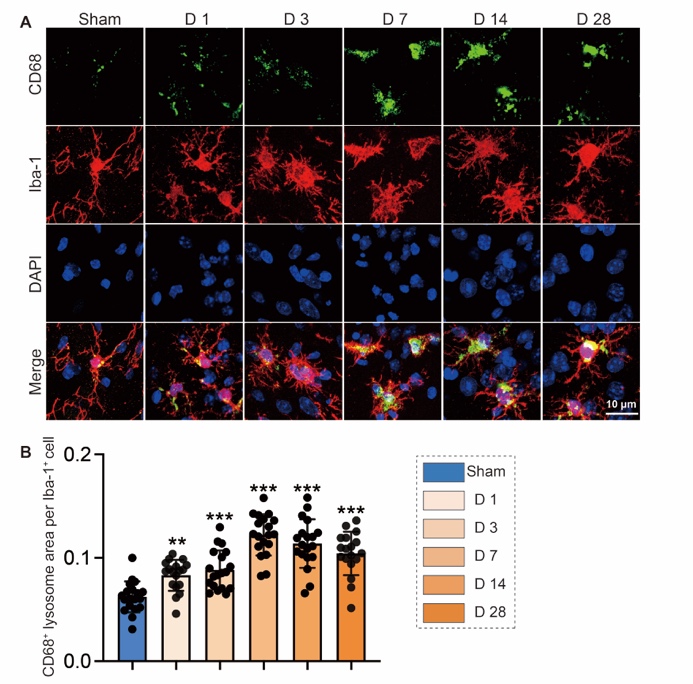


**Figure S3 Microglial phagocytosis ability after tMCAO.** A. CD68^+^ lysosome content (green) in Iba-1^+^ microglia (red) in the ischemic penumbra of sham and tMCAO mice. Scale bars, 10 μm. B. Quantification of the lysosomal area relative to the microglial area. Statistics are derived from 18-19 slices, n = 6 mice per group. Significance was calculated using one-way ANOVA, Tukey’s multiple comparisons test. Data are presented as mean ± SD; **p* < 0.05, ***p* < 0.01, ****p* < 0.001.

**
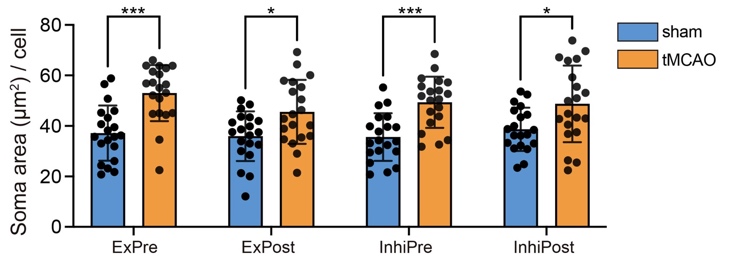
**

**Figure S4** **Microglial activation after ischemic stroke.** The soma area of Iba-1+ cell bodies (each symbol indicates one cell) in the ischemic penumbra. Statistics are derived from 12 slices, n = 4 mice per group. Significance was calculated using two-tailed unpaired Student’s *t* test. Data are presented as mean ± SD. **p* < 0.05, ****p* < 0.001.


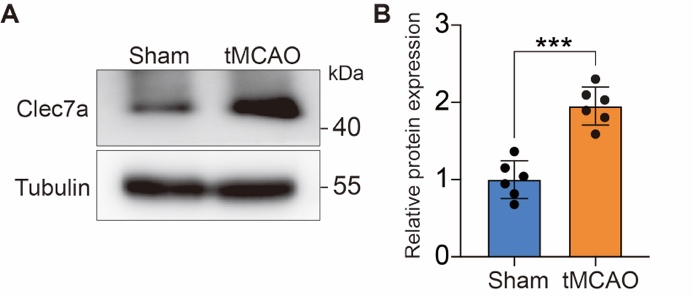


**Figure S5 Increased Clec7a expression level after tMCAO.** Representative western blot images (A) and quantification (B) of Clec7a protein level in the ischemic brain hemisphere on day 7 after tMCAO. n = 6 mice per group. Significance was calculated using a two-tailed unpaired Student’s *t* test. Data are presented as mean ± SD; ****p* < 0.001.


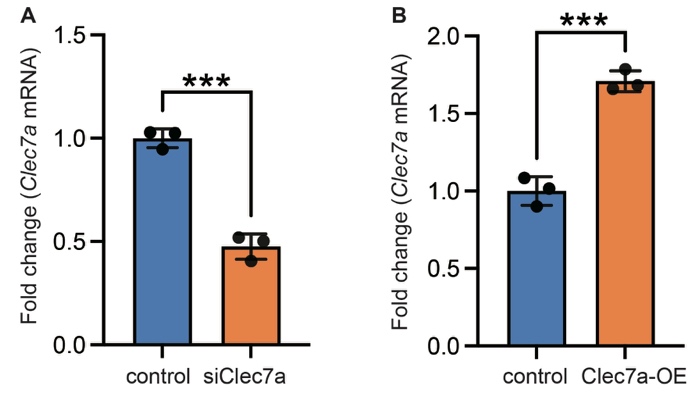


**Figure S6.** **Verification of Clec7a expression.** A. qRT‒PCR analysis showing the expression of Clec7a in BV2 cells at 48 h following siRNA transfection. B. qRT‒PCR analysis showing the expression of Clec7a in BV2 cells after transfection with the Clec7a overexpression lentivirus or the empty vector. n = 3 experiments. Significance was calculated using two-tailed unpaired Student’s *t* test. Data are presented as mean ± SD. ****p* < 0.001.


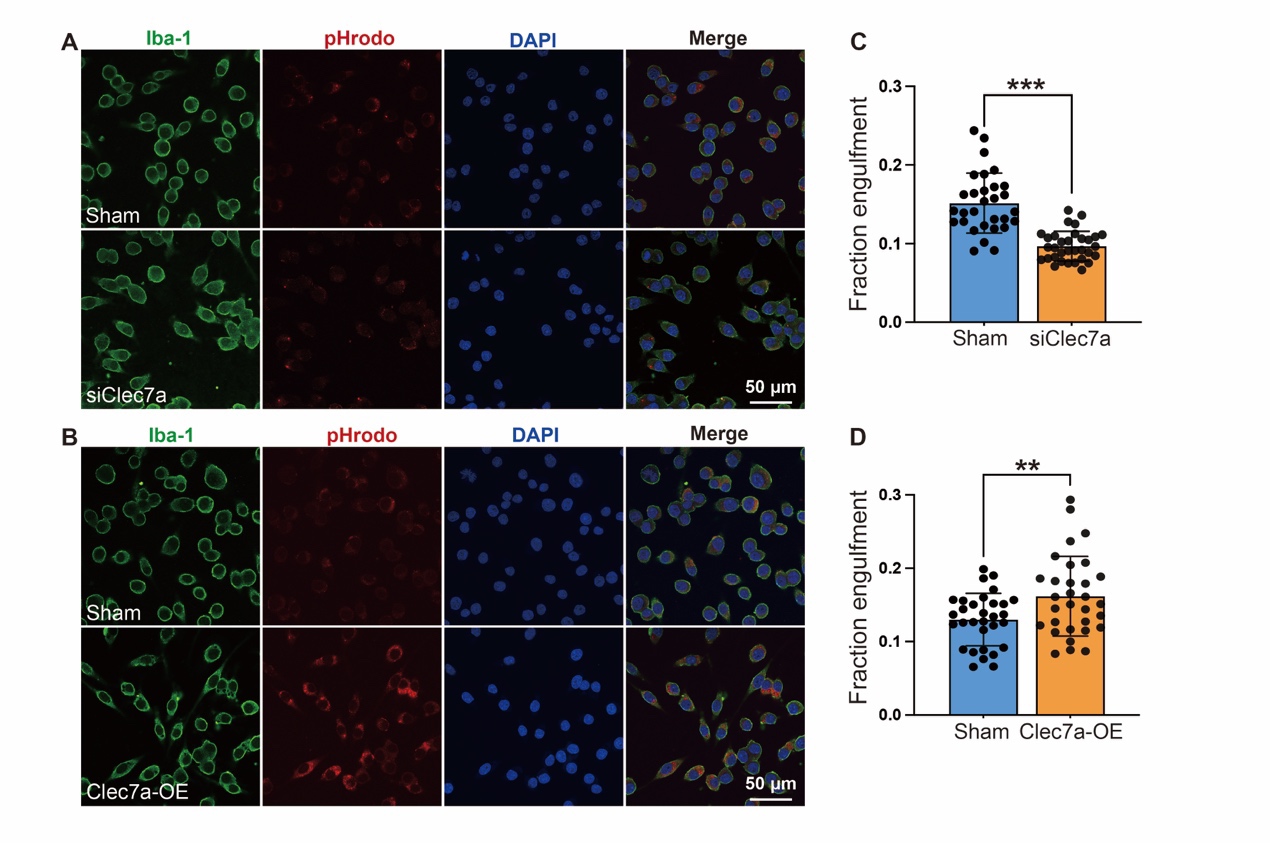
**Figure S7.** **Clec7a promotes microglia-mediated synaptic phagocytosis in vitro**. A. Representative images of cultured control or siClec7a BV2 cells engulfing pHrodo-conjugated synaptosomes. Scale bars, 50 μm. B. Representative images of cultured control or Clec7a-OE BV2 cells engulfing pHrodo-conjugated synaptosomes. Scale bars, 50 μm. C. Quantitative analysis of the pHrodo intensity in control or siClec7a BV2 cells. D. Quantitative analysis of the pHrodo intensity in control or Clec7a-OE BV2 cells. Statistics are derived from 18 slices, n = 9 per group. In C and D, significance was calculated using two-tailed unpaired Student’s *t* test. Data are presented as mean ± SD. ***p* < 0.05, ****p* < 0.001 vs. the sham group.


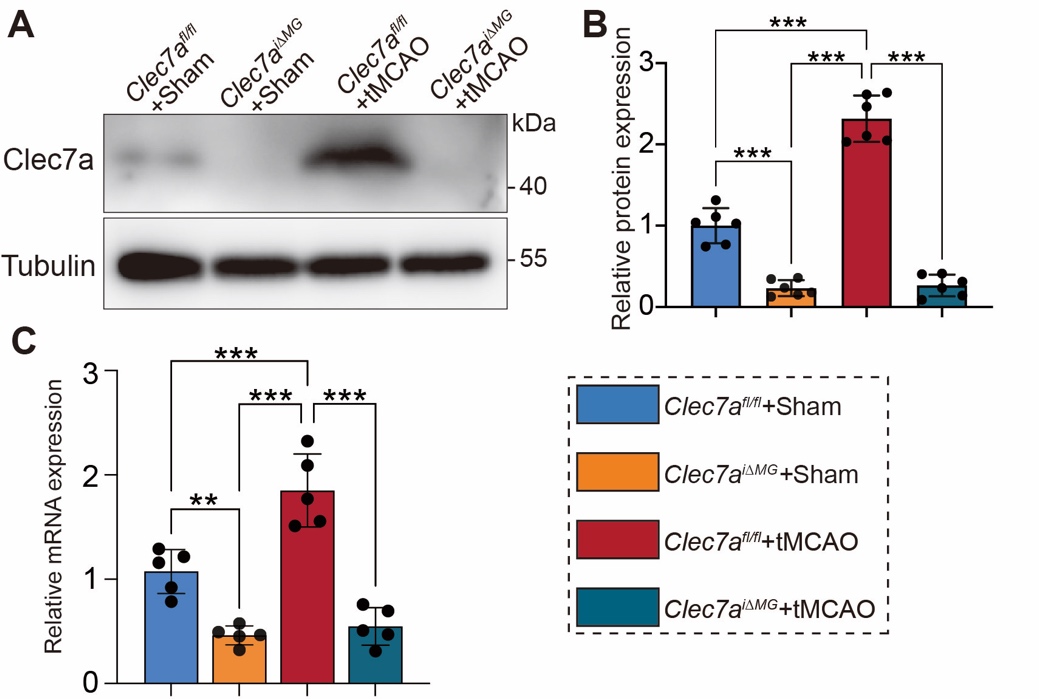


**Figure S8 Inducible knockdown of microglial Clec7a decreased Clec7a expression level after tMCAO.** (A–B) Representative western blot images (A) and quantification (B) of Clec7a expression in the ischemic brain hemisphere on day 7 after tMCAO. n = 6 mice per group. C. Relative transcription levels of Clec7a in the ischemic brain hemisphere on day 7 after tMCAO was determined by qRT-PCR. n = 5 mice per group. In B and C significance was calculated using one-way ANOVA, Tukey’s multiple comparisons test. Data are presented as mean ± SD. ***p* < 0.01, ****p* < 0.001.


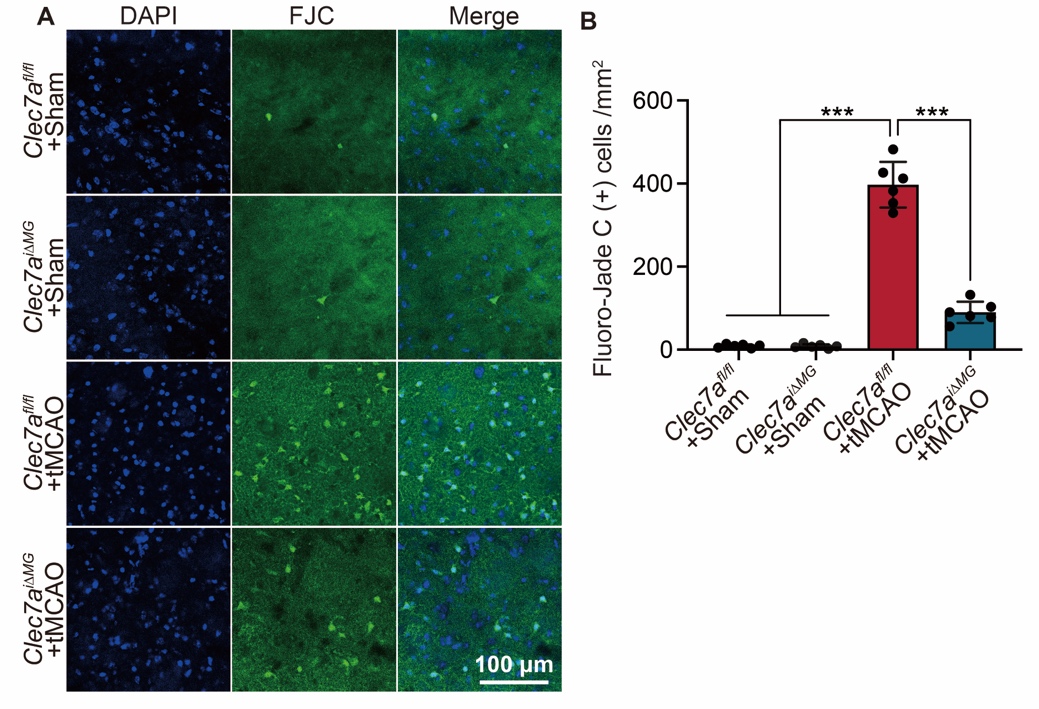


**Figure S9 Clec7a alleviates neuronal injury after tMCAO.** Representative confocal images (A) of FJC staining and quantitative analyses (B) in the ischemic penumbra of sham and tMCAO mice. n = 6 mice per group. Scale bars, 100 μm. In B significance was calculated using one-way ANOVA, Tukey’s multiple comparisons test. Data are presented as mean ± SD. ****p* < 0.001.


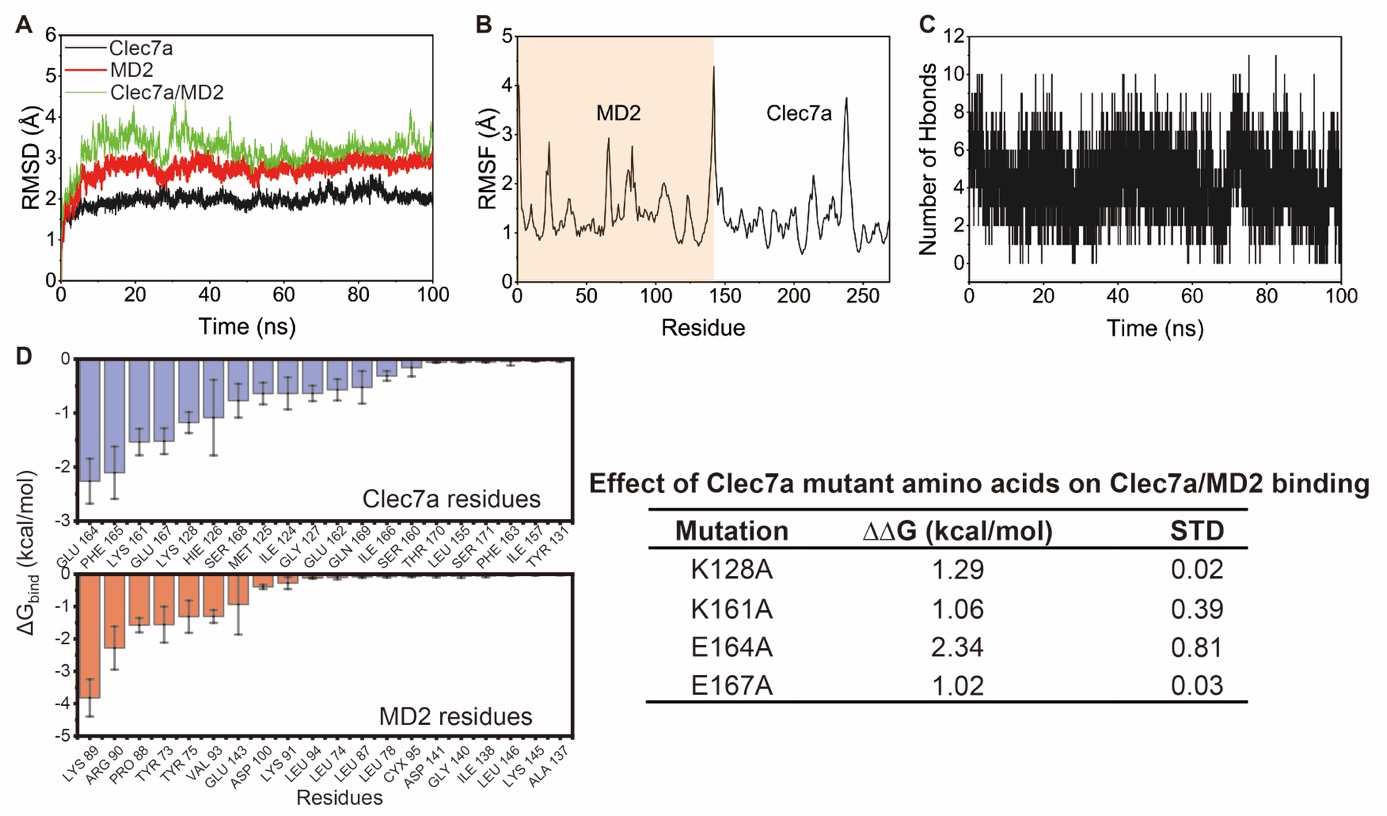


**Figure S10 The stability of Clec7a interact with MD2 in molecular dynamics simulation.** A. The root-mean-square deviation (RMSD) value during MD simulation. B. The root-mean-square fluctuation (RMSF) value during molecular dynamics (MD) simulation. C. During MD simulation, the number of hydrogen bonds between small molecules and proteins changed. D. The energetic contribution of amino acids affecting Clec7a/MD2 binding.


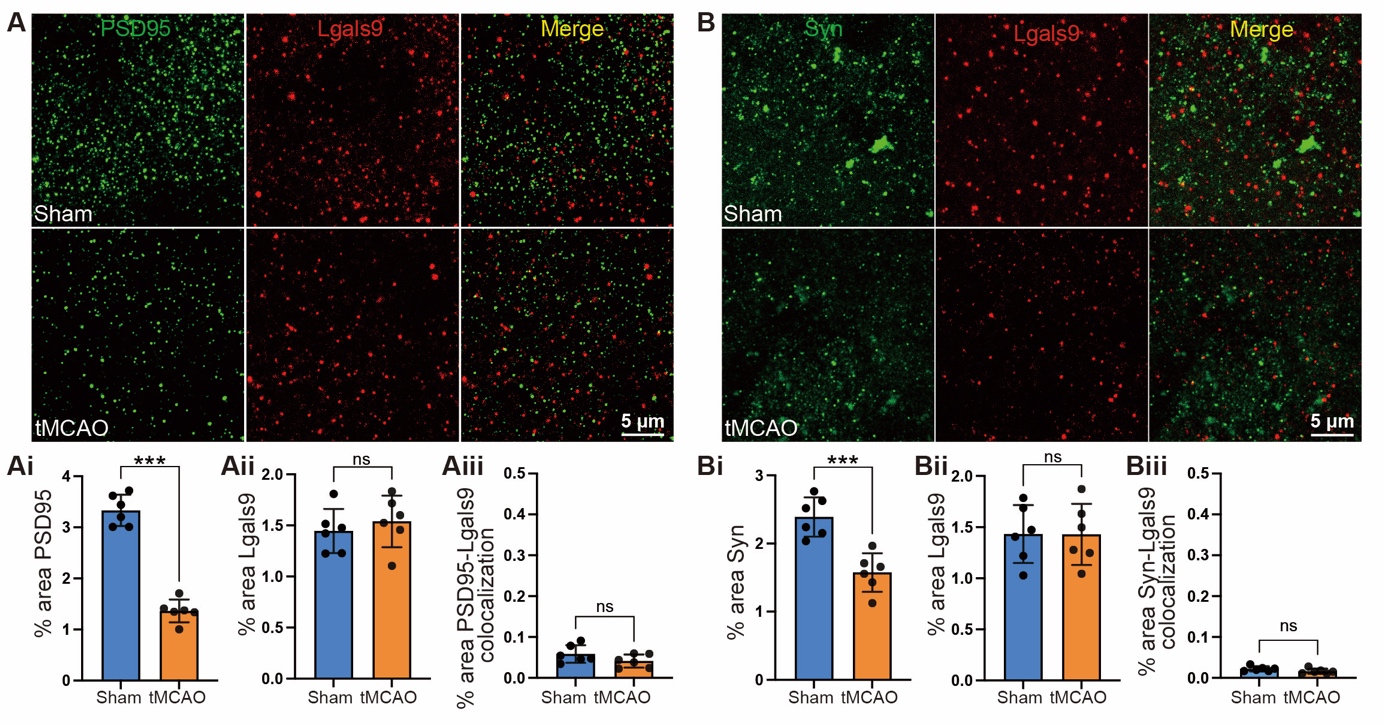


**Figure S11 Lgals9 not localizes to synapses in Sham and tMCAO mice.** (A–B) Representative confocal images in the ischemic penumbra of sham and tMCAO mice immunostained against Lgals9 and postsynaptic PSD95 (A) or presynaptic Syn (B). Quantification of total PSD95 (Ai), total Syn (Bi), and total Lgals9 (Aii and Bii) fluorescence area and colocalization of Lgals9 with PSD95 (Aiii) or Syn (Biii) in the ischemic penumbra of sham and tMCAO mice. Statistics are derived from 18 slices, n = 6 mice per group. Scale bars, 5 μm. Significance was calculated using two-tailed unpaired Student’s *t* test. Data are presented as mean ± SD. ****p* < 0.001, ns indicates no significant difference.


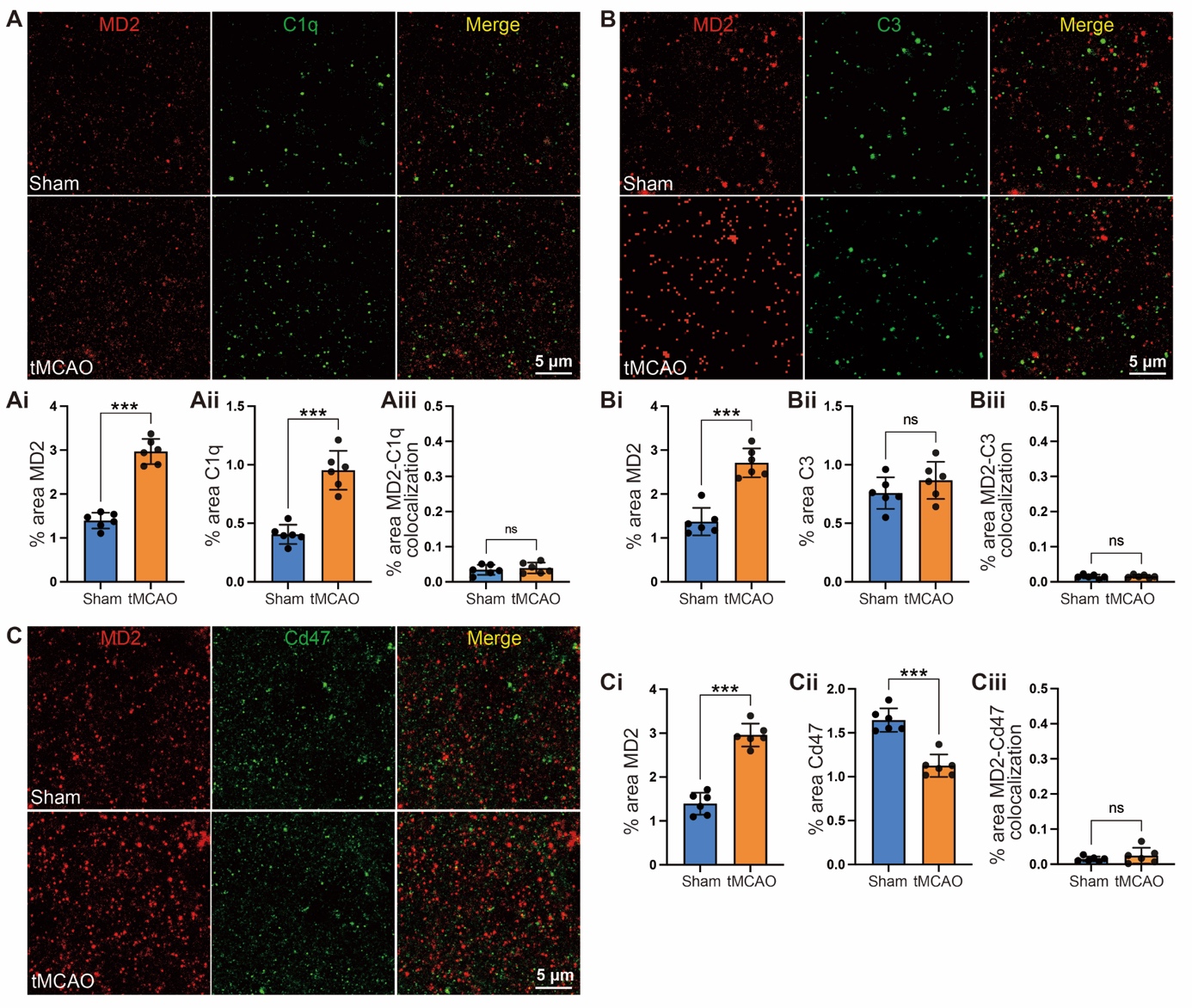


**Figure S12 MD2 not localizes to C1q, C3, Cd47 in Sham and tMCAO mice.** (A–C) Representative confocal images in the ischemic penumbra of sham and tMCAO mice immunostained against C1q (A) C3 (B) or Cd47 (C) and MD2. Quantification of total MD2 (Ai, Bi and Ci), total C1q (Aii), total C3 (Bii) and total Cd47 (Cii) fluorescence area and colocalization of MD2 with C1q (Aiii), C3 (Biii) or Cd47 (Ciii) in the ischemic penumbra of sham and tMCAO mice. Statistics are derived from 18 slices, n = 6 mice per group. Scale bars, 5 μm. Significance was calculated using two-tailed unpaired Student’s *t* test. Data represent mean ± SDs. ****p* < 0.001, ns indicates no significant difference.
